# A Novel Ruthenium-Fluvastatin Complex Downregulates SNCG Expression to Modulate Breast Carcinoma Cell Proliferation and Apoptosis via Activating the PI3K/Akt/mTOR/VEGF/MMP9 Pathway

**DOI:** 10.1155/2021/5537737

**Published:** 2021-06-07

**Authors:** Wei Liang, Junfeng Shi, Haiyan Xia, Xiaowei Wei

**Affiliations:** Department of Oncology, Nanjing First Hospital Nanjing Medical University, Nanjing 210006, China

## Abstract

Breast cancer is the most common cause of malignancy and cancer-related morbidity and death worldwide that requests effective and safe chemotherapy. Evaluation of metallodrug-based anticancer agents and statins as chemotherapeutics with fewer side effects is a largely unexplored research field. Synthesis and characterization of the ruthenium-fluvastatin complex were achieved using multiple spectroscopic techniques and thus further examined to evaluate its chemotherapeutic prospects in both MDA-MB-231 and MCF-7 cancer lines and eventually in vivo models of DMBA-induced mammary carcinogenesis in rodents. Our studies indicate that the metal and ligand chelation was materialized by the ligand's functional groups of carbonyl (=O) oxygen and hydroxyl (-OH), and the complex has been observed to be crystalline and able to chelate with CT-DNA. The complex was able to reduce cell proliferation and activate apoptotic events in breast carcinoma cell lines MCF-7 and MDA-MB-231. In addition, the complex was able to modify p53 expressions to interfere with apoptosis in the carcinoma of the breast, stimulated by the intrinsic apoptotic path assisted by Bcl2 and Bax in vivo, yet at the same point, controlling the PI3K/Akt/mTOR/VEGF pathway, as obtained from western blotting, correlates with the MMP9-regulated tumor mechanisms. Our research reveals that ruthenium-fluvastatin chemotherapy may disrupt, rescind, or interrupt breast carcinoma progression by modifying intrinsic apoptosis as well as the antiangiogenic cascade, thereby taking the role of a potential candidate in cancer therapy for the immediate future.

## 1. Introduction

Breast cancer in women is the most recurrent cancer in any of the five continents diagnosed worldwide, with an approximate 2.1 million new cases in 2018 [1]. The incidence of breast cancer in the Asian countries is still underneath as compared to that in Europe or America, but Asia's contribution in the worldwide burden of breast cancer is speedily escalating as a result of expressed economic growth and urbanization [2]. The situation in China has been worrying partly because of its fast population growth and socioeconomic progress. As a densely populated country, China accounts for one-fourth of total cancer-related death, and there has been an increasing incidence in cancer prevalence, mortality rate, and emergence among young people [3]. Breast cancer has often been diagnosed as an invasive malignant tumor without curable therapy [4]. Regretfully, the molecular mechanistic pathway underlying breast cancer is still unclear. Hence, a good understanding of the genetic underpinnings of breast cancer has great importance, and the alteration from researchers for further in-depth study was sought.

Current treatment strategies have limitations which lead to clinical resistance and morbidity associated with therapy owing to their adverse effects and limited efficacy on tumors. Novel molecules are therefore urgently required to combat emerging cases of breast cancer with less adverse effects, reduced tumor recurrence, and reduced mortality. An interesting approach called drug repurposing in the one hand and drug combination approach in another hand to tackle this problem are progressively being investigated and applied [5, 6]. By addressing the significance, the global market for drug repurposing almost touched nearly 20.7 billion euro in 2015 and is expected to reach 26.6 billion euro by 2020 [7]. Because of benefits such as reduced toxicity, improved effectiveness, reduced dose at an equivalent or enhanced level of effectiveness, and counter drug resistance, drug combination represents an impressive and progressively used approach which has become a standard for treating cancer [6]. Particularly in cancer, various clinical trials are conducted with a crescent focus on the combination of cytotoxic drugs [8]. The combination of repurposed pharmaceutical agents with other chemotherapeutic agents continues to show impressive outcomes which are beneficial when traditional monotherapy for cancer patients has failed to provide safe and tolerable treatment [9]. To overcome these challenges, our work is oriented towards investigating the effect of drugs like statins that can be repurposed for breast cancer therapy, in combination with chemotherapeutic transition metal like ruthenium.

Statins elicit several effects beyond their lipid-reducing roles defined as the pleiotropic effects [10] often caused by blocking the prenylation of a multitude of intracellular signaling proteins, thus affecting the development of cancer among statin users [11–15]. Statin has been delineated to be associated with a reduced rate of cancer progression and cancer-associated mortality [16, 17] from gastrointestinal carcinoma [18], breast carcinoma [19], hepatocellular carcinoma [20], prostate cancer [21], lung adenocarcinoma [22], and pancreatic carcinoma [23]. Fluvastatin is one of the members of the statin family, broadly used as a lipid lowering agent. In addition to its cholesterol-reducing activities, fluvastatin exhibited the anticancer activities by inducing apoptosis in glioma, breast, and hepatocellular carcinoma cell lines [24–26]. Notably, fluvastatin was shown to have a chemoadjuvant effect on experimental pancreatic cancer [27].

Along with platinum-based cancer drugs, several efforts have been made to design compounds based on ruthenium, since these compounds have been reported to have a lower number of side effects due to their alternate modes of action [28]. Ruthenium complexes have in many cases exhibited strong cytotoxicity against platinum-resistant tumor cell lines, rendering them as exemplary targets for further investigation [29, 30]. It may be acknowledged that many ruthenium-containing compounds including RAPTA-C [31], NAMI-A, and KP1019 [32] have officially managed to reach clinical trials for diverse therapeutic carcinoma management.

Despite advancement in the treatment, the high recurrence rate of metastasis is associated with poor prognosis of endocrine-related cancer [33]. Thus, a better understanding of the molecular mechanism underlying breast cancer proliferation and metastasis is necessary to identify the potential target for effective therapy. Synucleins are ubiquitously expressed in neuronal cells and abundantly present in presynaptic terminals and have also been involved in nonneural diseases, especially hormone-responsive breast cancers [34]. Numerous reports have also suggested that SNCG is not expressed in normal and benign breast tissues but expressed abnormally in a high percentage of advanced and metastatic cancer [35] and stimulates hormone-dependent growth of breast cancer cells both in vitro and in nude mice [36]. Research aimed at elucidating the molecular mechanism that triggers the oncogenic function of this protein reported that SNCG expression in the breast cancer cells has developed a more malignant phenotype with increased motility of the cells [34], intensified the activity of transcriptional factor of ER*α* [36], enhanced resistance to antimicrotubular drugs, and expedited the microsomal instability [37]. Cumulative results indicate that SNCG is a novel unfavorable prognostic marker for the development of breast cancer and a possible target for the treatment of breast carcinoma. On the basis of this data, the current research uses molecular docking, a widely used bioinformatics technique to evaluate how the molecule of ruthenium-fluvastatin binds and interacts with the SNCG target proteins. These in silico methodologies aid towards classifying drug targets through computer-assisted designing that helps to (i) envision specific active sites for evaluating target structures, (ii) produce potential molecules that identify the target, (iii) determine their comparative receptor binding affinities, and (iv) modify molecules to optimize binding abilities [38]. AutoDock is one of the many software programs that consists of a series of automatic docking techniques developed to evaluate how molecules attach to a particular targeted protein with a preordained arrangement [39]. Therefore, our objective is to establish the optimum binding energies of the docked molecules and also to determine the ligand's binding position at the protein binding domain.

A combination of cell proliferation and apoptosis regulates the development of normal breast tissues. Conclusive evidence states that development of cancer is not only a manifestation of unregulated proliferative status along with impaired apoptosis [40, 41]. In the context of breast cancer, the Bcl2 gene and the tumor suppressor like p53 genes are extensively researched [42, 43]. Bax and Bcl2 are indeed the key proapoptotic modulators which could additionally improve the PI3K/AKT-related cascade and other pathways related to cell survival and death [44]. Furthermore, natural agents may also affect cell migration and invasion of tumor cells by modulating the matrix metalloproteinases (MMPs). Between others, MMP9 is of particular interest, as patients with increased MMP9 expression have been identified to end up with poorer diagnosis [45]. In addition, breast cancer development includes genetic variations of p53, modified by the angiogenic pathway (VEGF) and mTOR-related signaling pathway, and proapoptotic protein such as Bax correlated with the increased formation of antiapoptotic protein Bcl2 and proliferating cell nuclear antigen (PCNA) [46, 47].

7,12-Dimethylbenz (*α*) anthracene may be the most harmful polyaromatic hydrocarbon with the widest environmental application [48]. DMBA metabolizes and produces various reactive metabolic intermediates during the carcinogenic cycle, by which stable formation of DNA adducts which are genotoxic and mutagenic initializes carcinogenic events, close to those in humans [49]. The oxidative imbalance influences different types of proteins and genes that modify multiple signaling cascades such as angiogenic process, apoptotic events, cell formation, repairing of DNA, proliferative events, and invasion [50, 51].

Taking all of it into account, we hypothesized and investigated by well-designed experiments in this current study that ruthenium-fluvastatin could interact with cellular proliferation and induce cell apoptosis by modulating the apoptotic regulators and downstream events such as caspase cascade thus inhibiting the invading and migrating properties of cancer cells. Chemotherapeutic actions in the breast cancer paradigm and the underlying mechanistic approach of the ruthenium-fluvastatin complex have not yet been extensively analyzed. This study delineates the synthesis of the novel ruthenium-fluvastatin complex along with characterization of its chemical structure and further investigates the chemotherapeutic efficacy against mammary cancer in both in vitro and in vivo studies.

## 2. Materials and Methods

### 2.1. Chemicals and Reagents

The chemicals used for experimental analysis is of pure analytical category. Extra pure methanol, fluvastatin ((E,3R,5S)-7-[3-(4-fluorophenyl)-1-propan-2-ylindol-2-yl]-3,5-dihydroxyhept-6-enoic acid), TPTZ (2,4,6-tri(2-pyridyl)-s-triazine), ruthenium chloride (RuCl_3_ · *x*H_2_O), highly polymerized CT-DNA (calf thymus DNA), Tris-HCl, 7,12-dimethylbenz (*α*) anthracene (DMBA), foetal bovine serum (FBS), 3,3′-diaminobenzidine (DAB), proteinase K, insulin L-glutamine, sodium pyruvate, streptomycin, penicillin, ABTS (2,2′-azinobis 3-ethylbenzothiazoline-6-sulphonic acid diammonium salt), streptavidin peroxidase, MTT (3-(4,5-dimethyl thiazole-2-yl)-2,5-diphenyltetrazoliumbromide), DPPH (2,2-diphenyl-2-picrylhydrazyl), biotinylated goat anti-rabbit IgG, annexin V, and propidium iodide (PI) were purchased from Sigma-Aldrich Chemical Co. (St. Louis, MO, USA). Antibodies specific for p53, VEGF (627501), caspase-3, Akt, mTOR, PI3K, and pro- and active caspase-3 were acquired from BioLegend (Sun Diego, CA, USA). Rabbit anti-rat p53, Bax, Bcl2, Ki-67, MMP9, and goat anti-rabbit IgG secondary antibodies were bought from AnaSpec Inc. (San Jose, CA, USA). The MCF-7 and MDA-MB-231 breast cancer cell lines were obtained from the American Type Culture Collection, 10801 University Boulevard, Manassas, VA 20110, USA. Kits for detecting apoptosis were purchased from Takara Bio Inc. (Japan). Other chemical agents utilized for the research purpose were procured from local companies in the finest possible form.

### 2.2. Pharmacophore Analysis

#### 2.2.1. Target Protein Selection

The reviewed sequence of human gamma-synuclein was retrieved from the universal protein sequence database UniProt (http://www.uniprot.org/). The sequence of selected proteins was used to predict sequence similarities and to estimate the sequence template by PSI-BLAST. The specified sequence of a template was used to construct three-dimensional protein structures utilizing the Swiss model. Homology modeling using the Swiss PDB Viewer assists in envisaging the orientation of the protein structure and was validated to check the overall quality of protein and stereochemical activity of atoms and amino acids.

#### 2.2.2. Ligand Structure Design and Pharmacophore Analysis

Using the ACD/ChemSketch software, chemical structures were designed to attach all chemical compositions and the ultimate output was saved in MOL2 format. The training sets were used to determine the pharmacophore using the Molinspiration server, and QSAR properties were envisaged using HyperChem. A training set helps to predict the complex polarity and flexibility, to examine MM3 force fields, to examine the HOMO and LUMO, and to understand new molecular orbital of individual compounds. The scaffolds were identified, and then, we accelerated screening; a screened pool is focused on biotargets to inhibit the diseases. Structural screening, fragment analysis, and pharmacological analyses were used to screen the ligand based on the interaction with target apoptotic proteins.

#### 2.2.3. Molecular Docking

The AutoDock 4.6 software was used to predict protein-ligand exchange utilizing different factors such as preparing protein properties, adding Gaussian charges, adding hydrogen atoms with a polar amino acid zone, and planning a ligand molecule with rotatable angle bond interaction. Grid maps of different grid points, centered on the ligands of the complex structure, were used for receptors, respectively, to cover binding pockets. A set of the Lamarckian genetic algorithm was used for molecular docking simulations. Simulations were performed using up to 2.5 million energy evaluations with a maximum of 27000 generations. Each simulation was performed 10 times, yielding 10 docked conformations. The lowest energy conformations were regarded as the binding conformations between the ligands and the proteins. Further, the reverse validation processes ensured that the identified hits really fit the generated pharmacophore models and active sites of targets. All the parameters required for molecular docking and pharmacophore mapping were fixed as used in the regular process.

### 2.3. Synthesis of the Ruthenium-Fluvastatin Complex

**Figure 1:**
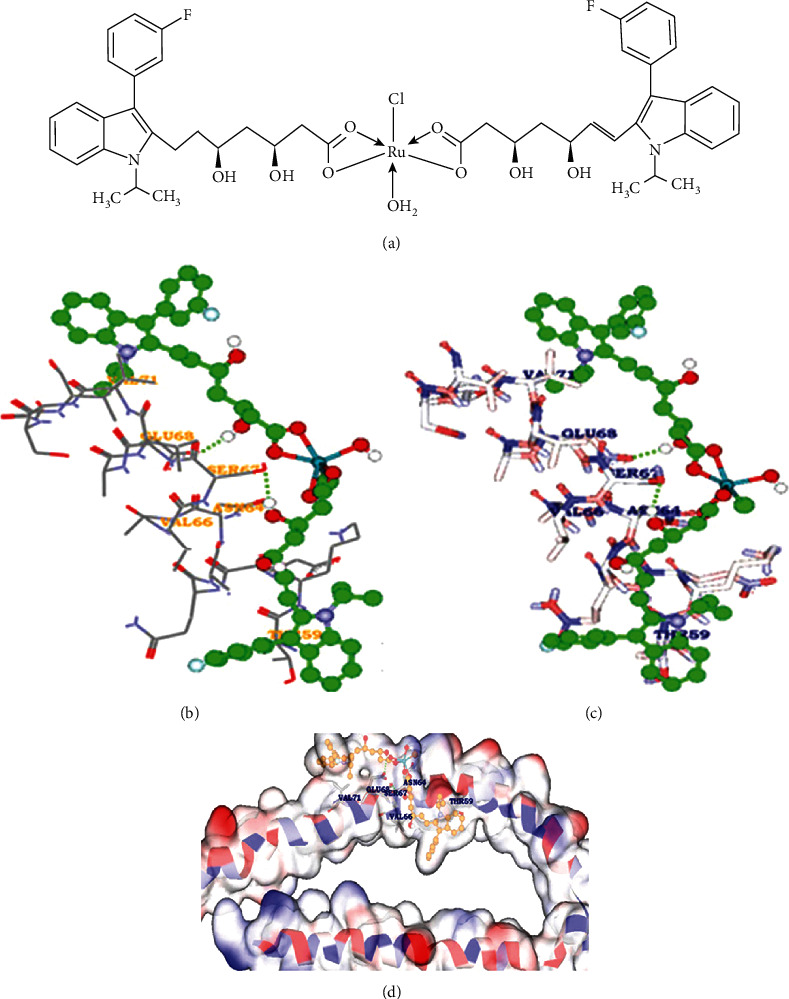
(a) The possible structure of the ruthenium-fluvastatin complex. (b–d) Receptor-ligand hydrogen bonding interactions of Ru-flu with active site residues of gamma-synuclein.

Approximately 433.45 mg (1 mmol) of fluvastatin sodium was dissolved in 60 mL HPLC analytical category ethanol at a room temperature of 27°C, with continuous mixing using a mechanical stirrer. In another conical flask, approximately 103.5 mg (0.5 mmol) of ruthenium chloride was dissolved in 40 mL of ethanol and added dropwise to the fluvastatin solution with continuous stirring for 24 hours. After complete mixing, the resultant solution was refluxed at 80°C for 3 hours. The reaction mixture was kept in a vacuum desiccator over silica gel for seven days. The obtained product was brown in color and was found to be soluble in ethanol and dimethyl sulfoxide (DMSO), with the percentage yield of the product being 87%.  Figure 1(a) represents the possible structure of the ruthenium-fluvastatin complex.

### 2.4. Characterizations of the Ruthenium-Fluvastatin Complex

The UV-visible spectra of the ruthenium-fluvastatin complex and fluvastatin were recorded via a UV-1800 Shimadzu double-beam spectrophotometer with a typical 1.00 cm quartz cell. FTIR spectroscopy (ALPHA-T, Bruker, Rheinstetten, Germany) was used to document the infrared spectrum of the complex over the span of 500-4000 cm^−1^ wavelength to evaluate the complexation by detecting the metal oxide bond. The molecular structure of the ruthenium-fluvastatin complex was studied by employing tandem mass spectrometry (ESI-MS) techniques with electrospray ionization. Molecular ions (*m*/*z*) were scanned over a span of 150-1100. A Bruker-Avance-600 MHz spectrometer was used for studying the ^1^H-NMR spectrum of the complex dissolved in DMSO. Tetramethylsilane (TMS) was used as an internal reference. Scanning electron microscopy (JEOL MAKE, UK; model: JSM6360) was used to examine the morphological appearance of the sample at an accelerating voltage of 17 kV. To examine the surface and morphological features, micrographs were recorded at different magnifications (60x, 200x, 500x, and 1000x). X-ray diffraction (XRD) of the complex was recorded by using an XPERT-PRO Diffractometer (PAN Analytical, Almelo, Netherlands) using X'celerator operating at 40 kV and 30 mA with the Bragg-Brentano geometry with a step size of 0.033(2*ɵ*) and step time of 19.43 s.

### 2.5. Assessment of Antioxidant Status of the Ruthenium-Fluvastatin Complex by the DPPH, FRAP, and ABTS Processes

#### 2.5.1. DPPH Assay

Free radical scavenging ability of the complex was assessed by the DPPH radical scavenging assay according to the procedure described by Lim and his coresearcher [52]. The hydrogen atom-donating ability of the complex was determined by the decolorization of methanol solution of 2,2-diphenyl-1-picrylhydrazyl (DPPH). DPPH produces violet/purple color in methanol solution which fades to shades of yellow color in the presence of antioxidants. Subsequently, 100 *μ*L of the complex as well as ligands at different concentrations (5, 10, 15, 20, and 25 *μ*M) was mixed with 0.1 mL of DPPH solution (0.2 mg/mL in ethanol), and the absorbance was measured at 515 nm every 5-minute interval and further assessed for 30 minutes (*A*_s_), whereas a solution containing only DPPH functioned as blank (*A*_c_). The percentage of radical scavenging action (RSA%) was calculated as

(1) $$\begin{array}{c} \left(\text{RSA}\%\right)=\frac{100\left({\text{A}}_{\text{c}}-{\text{A}}_{\text{s}}\right)}{{\text{A}}_{\text{c}}}, \end{array}$$

#### 2.5.2. FRAP Assay

The FRAP assay was carried out by the method of Benzie and Strain [53] as developed by Griffin and Bhagooli [54]. The working FRAP reagent was prepared by mixing 300 mM acetate buffer (pH 3.6), 10 mM 2,4,6-tripytidyl-s-triazine (TPTZ) solution, and 20 mM FeCl_3_·6H_2_O at a 10 : 1 : 1 ratio prior to use and heated to 37°C in a water bath. 3 mL of the FRAP reagent was added to 100 *μ*L of various concentrations (5-40 *μ*M) of the complex and ligands. Following reaction, light bluish coloration of the FRAP solution shifted to dark blue, and the change in absorbance was detected at wavelength 593 nm and expressed as mmol Fe^2+^/g of sample.

#### 2.5.3. ABTS Assay

The radical scavenging activity of the ruthenium-fluvastatin complex by the ABTS method has been evaluated utilizing the process outlined by Pennycooke and coworkers [55]. Following the incorporation of fluvastatin and the complex to the ABTS solution (incubated at room temperature for 10-12 minutes), the absorbance was taken at 734 nm. The equation below was used to measure the percentage of radical scavenging action (RSA%):

(2) $$\begin{array}{c} \text{Radical scavenging activity at }750\text{ nm }\left(\%\right)=1-\frac{A_{\text{f}}}{A_{0}}100, \end{array}$$

where *A*_0_ is the absorbance of free radical cation and *A*_f_ is the absorbance observed 10 min after incorporation of the complex.

### 2.6. DNA Binding Assay of the Ruthenium-Fluvastatin Complex

CT-DNA intercalation with the complex was calculated using a UV-visible spectrophotometer (UV-1800 Shimadzu) based on the technique recorded by Dehghan et al. [56]. The intrinsic binding constant was calculated as

(3) $$\begin{array}{c} \frac{\text{DNA}}{\varepsilon_{\text{a}}-\varepsilon_{\text{f}}}=\frac{\text{DNA}}{\varepsilon_{\text{b}}-\varepsilon_{\text{f}}}+\frac{1}{\text{Kb}\left(\varepsilon_{\text{b}}-\varepsilon_{\text{f}}\right)}, \end{array}$$

where DNA represents the number of base pairing of DNA, *ε*_a_ represents the extinction coefficient (*A*_obs_/Ru) factor, *ε*_f_ is the free drug-related extinction coefficient, and *ε*_b_ represents the bound drug-associated extinction coefficient, and the complex-associated calibration curve is derived from *ε*_f_ in the aqueous solution. *ε*_a_ represents the ratio of the recorded absorbance to the concentration of the complex by Beer's law.

### 2.7. *In Vitro* Experimentation

#### 2.7.1. Cell Culture

The breast cancer cell lines MCF-7 and MDA-MB-231 were acquired from the American Type Culture Collection (ATCC) (Manassas, VA, USA). The tumor cells were normally sustained in DMEM, enriched by 10% FBS (foetal bovine serum) constituting antibiotics such as penicillin/streptomycin (0.5 mL^−1^) in an environment of 5% CO_2_ and 95% air at 37°C.

#### 2.7.2. Cell Viability Assay

The experiment was assessed by using mitochondrial succinate dehydrogenase to metabolize tetrazolium salts MTT (3-(4,5 dimethylthiozol-2-yl)-2,5-diphenyltetrazolium bromide), which is yellow to produce formazan crystals. The MCF-7 and MDA-MB-231 cells were plated in a 5% CO_2_ humidified incubator and exposed to a number of ruthenium-fluvastatin doses for 24 hours, containing proper growing media in a 96-well plate containing 5.0 × 10^3^ cells for each well, and incubated nightly at 37°C. Upon treatment with 15, 35, 75, and 95 *μ*M concentrations of the complex for 24 hours, the medium was withdrawn and the MTT solution (0.5 mg/mL) was applied to each well and incubated at 37°C for the next 3 hours. On a microplate reader, the optical density of solubilized crystals in DMSO was estimated at 560 nm. The cell viability percentage was determined by the following equation.

(4) $$\begin{array}{c} \%\text{viability}=100-\%\text{of cytotoxicity}. \end{array}$$

The cell viability assay was performed in triplicate.

#### 2.7.3. Assessment of Apoptotic Cells by DAPI Staining

DAPI staining was used to analyze apoptotic morphology, i.e., nuclear chromatin condensation. In 6-well plates, MCF-7 and MDA-MB231 cells were seeded. After an overnight incubation, cells were treated with varying amounts of the complex for 24 hours. Cells were then extracted, fixed in 4% paraformaldehyde, treated with 0.25 percent Triton X-100 in TBS for 15 minutes at room temperature, and stained with 50% DAPI for 30 minutes at room temperature. Following PBS washing, samples were kept in the dark at 4°C and studied under a fluorescence microscope in three separate independent experiments. [57].

#### 2.7.4. Clonogenic Assay

The inhibitory effect of the ruthenium-fluvastatin complex on MCF-7 and MDA-MB-231 cells on proliferation was determined by the clonogenic assay. The cells were trypsinized to create a single-cell suspension and implanted in six-well plates at a density of 500 cells/well in 2 mL of medium supplemented with 10% FBS, kept in a humidified compartment having an atmosphere of 95% air and 5% CO_2_ at 37°C. After 24 hours of incubation, culture was replaced with fresh media containing three different concentrations of the ruthenium-fluvastatin complex at 30, 70, and 100 *μ*M in MCF-7 cells and 50, 75, and 200 *μ*M in MDA-MB-231 cells along with 2% FBS and cultured for two weeks. After 2 weeks, the cell culture medium was removed and the cells were thoroughly washed with PBS. Cell fixation was done by using 100% methanol kept at -20°C for 30 minutes. Colonies were stained with 0.5% crystal violet in 25% (*v*/*v*) methanol for 1 hour at room temperature. The excess dye was removed by gently rinsing with moderate water flow for 15 minutes. Following washing and drying, the colonies were visually counted to contain >50 cells/colony. The clonogenicity was measured by the help of the equation below:

(5) $$\begin{array}{c} \text{Clonogenicity}=\left(\frac{\text{Cloning number}}{500x}\right)\times 100. \end{array}$$

The clonogenic assay was performed in triplicate.

#### 2.7.5. Apoptotic Assay by Flow Cytometry and Cell Cycle Analysis

MCF-7 and MDA-MB-231 cells were cultured in a 6-well plate at a density of 0.5 × 10^6^ cells/3 mL and incubated in a carbon dioxide environment overnight at 37° C for 24 h. At the end of 24 h, MCF-7 cells were treated with 30, 70, and 100 *μ*M concentrations of the complex and MDA-MB-231 cells were treated with 50, 75, and 200 *μ*M concentrations of the complex followed by incubation for the next 24 h. At the end of the treatment, the cells were harvested with trypsin-EDTA and fixed in 70% cold ethanol. Then, the cells were treated with an FITC-conjugated annexin V antibody followed by incubation in the dark. After further washing, the cells were treated with propidium iodide and then analyzed in a FACS analyzer. The assay was performed in triplicate.

#### 2.7.6. Caspase-3 Protein Detection by Flow Cytometry

MCF-7 and MDA-MB-231 cells (5 × 10^5^ cells/well) were cultured on a twelve-well plate and incubated at 37°C in a humidified atmosphere with 5% CO_2_ for 24 hours and subsequently treated with three concentrations of the ruthenium-fluvastatin complex for 24 hours. The cells were again washed properly with ice-cold PBS and resuspended with BD Cytofix/Cytoperm Solution (51-6896KC, BD Pharmingen) 400 *μ*L. The method was initiated by determining the quantity of BD Perm/wash buffer (51-6897KC, BD Pharmingen), and 20 *μ*L of rabbit anti-active caspase-3 polyclonal antibody (351-68655X, BD Pharmingen) was taken, so that each and every individual test was comprised of 100 mL of BD Perm/wash buffer and 20 *μ*L of anti-active caspase-3 antibody. After incubation on ice for 20 minutes, followed by centrifugation and washing with BD Perm/wash buffer, subsequently, BD Perm/wash buffer was further added followed by incubation with the antibody for thirty minutes at room temperature. Each and every individual tube was further rinsed with 1 mL of BD Perm/wash buffer, centrifuged, and then finally added with 300 *μ*L of BD Perm/wash buffer and analyzed by flow cytometry (BD Accuri C6 Plus flow cytometer). Values thus obtained were processed by using the FlowJo software. The caspase-3 assay was performed in triplicate.

#### 2.7.7. Detection of Akt, mTOR, P13K, VEGF, Procaspase-3, and Active Caspase-3 Protein Expression by Western Blot

Western blot analysis detected the expressions of Akt, mTOR, P13K, VEGF, procaspase-3, and active caspase-3 in cells MCF-7 and MDA-MB-231. The cells were treated for 24 hours after a medium change with three different doses of the ruthenium-fluvastatin complex and maintained for 6 hours. Cell lysates were extracted, and equivalent protein amounts were analyzed using SDS-PAGE electrophoresis, followed by a shift to a PVDF (polyvinylidene difluoride) membrane, and afterwards blocked to Tris buffer (25 mM) comprising 0.15 M NaCl, 0.1 percent around 20, and 2-5 percent nonfat dry milk. At 4°C, the membranes were cultured with the primary antibodies for Akt, mTOR, P13K, VEGF, procaspase-3, and active caspase-3 supplemented with a secondary antibody marked with horseradish peroxidase for 1 h. A chemiluminescent (ECL western blotting) kit was then used to recognize protein loading against *β*-actin [58]. All experiments were carried out in triplicate.

### 2.8. *In Vivo* Experimentation

#### 2.8.1. Animal Husbandry and Maintenance

Sprague-Dawley rats (120-125 g) of both sexes and 28-day Sprague-Dawley female rats (80-100 grams) were purchased from Nanjing Medical University, Nanjing, China, and quarantined even days before experimentation. Animals were housed in a 12-hour light/dark period in polypropylene containers and at 22°C (±3°C) at room temperature and nearly 50-58% humidity. Each animal was fed a semipurified basal diet and demineralized water ad libitum. The entire animal research process was performed in conjunction with the permission of Nanjing Medical University's Animal Ethics Committee and the Government's Regulatory Body (IACUS-1912129).

#### 2.8.2. Toxicological Studies

*(1) Acute Oral Toxicity Study (LD50)*. Acute toxicity evaluation of the ruthenium-fluvastatin complex was carried out by incorporating the recommendations for research on chemicals by the Organization for Economic Cooperation and Development (OECD), TG 420 (adopted in December 2001), to establish the LD_50_ values of the complex. Thirty numbers of Sprague-Dawley rats of both sexes (nulliparous and nonpregnant; 120 ± 5 g) were identified and allocated in five groups (six animals per group, three of each sex) plus control (dispensed in 0.5% carboxy methyl cellulose prepared as a carrier in drinking water at a dosage of 10 mL/kg body weight) and study groups (2000, 800, 600, 300, and 100 mg/kg ruthenium-fluvastatin complex). The rats were allowed food and supplies immediately after drug administration and placed under three-day surveillance [59].

*(2) Subacute Toxicity Studies*. Sprague-Dawley rats, both male and female (120 ± 5 g), were arbitrarily paired to four experimental groups: complex (25, 50, 100, and 200 mg/kg) and vehicle control groups. Each unit was composed of 10 rats, 5 per gender. For further hematology, serum biochemistry, and histological experiments, the animals were orally administered with the ruthenium-fluvastatin complex and sacrificed at the 28^th^ day by ether anesthesia.

#### 2.8.3. Histopathological Study of Rat Organs

Primary organs such as the liver, kidney, stomach, and testis were harvested from each experimental animal after 28 days of analysis and stored in 10% formalin solution. Tissue was drained by graded alcohol and preserved in paraffin wax at a low melting point on a 5-micron glass slide. By using xylene, the sections were deparaffinized and rehydrated by graded alcohol and subsequently stained with hematoxylin and eosin (H&E) for microscopic examination.

#### 2.8.4. *In Vivo* Experiments

*(1) Experimental Protocol*. After acclimatization, the animals were grouped into seven designated units such as I, II, III, IV, V, VI, and VII and each unit consisted of six animals. Once all the animals were 50 days old, DMBA was given in an oil emulsion as a single tail vein injection to groups II to IV at a dosage of 0.5 mg per 100 g body weight. The description below denotes the experimental layout of the groups.

Group I: animals constituted the normal untreated controls and received basal diet throughout experiment. Group II: these are comprised of carcinogen- (DMBA) treated animals. Group III: carcinogen- (DMBA) induced animals were treated with the Ru-fluvastatin complex (25 mg/kg body weight). Group IV: carcinogen- (DMBA) induced animals were treated with the Ru-fluvastatin complex (50 mg/kg body weight). Group V: carcinogen- (DMBA) induced animals were treated with the Ru-fluvastatin complex (100 mg/kg body weight). Group VI: carcinogen- (DMBA) induced animals were treated with ruthenium (50 mg/kg body weight). Group VII: carcinogen- (DMBA) induced animals were treated with fluvastatin (50 mg/kg body weight). Following 16 weeks of treatment, the animals were sacrificed in light ether anesthesia from each group preceded by midline incision from the pubis to the submaxillary region. Dissection of the skin was undertaken to reveal the six sections of mammary glands.

*(2) Histopathology of Mammary Tissue*. Ten animals were randomly selected from each individual group, excising the thoracic and abdominal inguinal mammary tissue from rats anesthetized with ether. Part of the breast tissue was set in 10% neutral buffered formalin, carefully washed, paraffin-coated, sliced into 5 *μ*m thick segments, and mounted on slides and subsequently treated with hematoxylin and eosin for histopathological investigations (H&E). All experiments were performed in triplicate.

*(3) Antioxidant Assay of Mammary Tissues*. Mammary tissue was crushed and homogenized (10 percent *w*/*v*) in 0.1 M phosphate buffer (pH 7.0) and centrifuged for 10 min, and thus, the generated supernatant had been used for the measurement of enzymatic antioxidants. The catalase reaction was analyzed using the method defined by Sinha and his associates [60]. The absorbance was evidenced at 620 nm; the CAT action was reported as *μ*mol of H_2_O_2_/min/mg protein consumed. Superoxide dismutase activity was assessed using the Awasthi technique [61]. This activity was expressed as units/min/mg protein. The GPx evaluation was carried out using the process provided by Rotruck et al. [62]. The activity was calculated as *μ*mol of GSH consumed/min/mg protein. All experiments were performed in triplicate.

*(4) Immunohistochemical Analysis of Mammary Tissue*. The tissues coated with formalin and set in paraffin are sliced into 5 *μ*m thickness to position on the glass slides and deparaffinized together with immersion in H_2_O_2_. For 1 hour, the segments were coated with goat serum, preceded by exposure to anti-mouse p53, Bcl2, Bax, and MMP9 antibodies (1 : 50 ratio) and maintained overnight at 4°C. The slides were immersed with PBS and subsequently cultivated for about 30 min with the HRP-conjugated secondary antibody streptavidin biotin and subsequently treated with DAB to the segments and counterstained with hematoxylin. The labeling index was measured as the number of positively stained cells with p53, Bcl2, Bax, and MMP9 to the total cell count. All experiments were performed in triplicate.

*(5) Cell Proliferating Assay*. The tissues coated with formalin and set in paraffin are sliced into 5 *μ*m thickness to position on the glass slides and deparaffinized accompanied by submersion in H_2_O_2_. The segments were covered with goat serum for 1 hour and exposed to the anti-mouse Ki-67 antibody at 4°C overnight. At room temperature, the positive test slides were processed for 30 min with the streptavidin biotin horseradish peroxidase complex. Tissues were treated with DAB (3,3′-diaminobenzidine) and hematoxylin [63]. All experiments were performed in triplicate.

*(6) TUNEL Assay of Mammary Tissues*. Tissues fixed in formalin, implanted in paraffin, and coated with poly-L-lysine were screened for 15 minutes in proteinase K solution (20 *μ*g/mL in PBS) and washed with water distilled twice. The tissues were then soaked with H_2_O_2_ (2% in PBS) at room temperature for 5 min, accompanied by treatment with the terminal deoxynucleotidyl transferase (TdT) buffer (30 mM Trizma base, pH 7.2, 140 mM sodium cacodylate, and 1 mM cobalt chloride) accompanied by a TdT reaction solution containing TdT and dUTP at 37°C for 90 min; 2 percent of the normal saline citrate was then added to the tissues (10 min) at room temperature to interrupt the reaction. After washing with PBS, the tissue segments were soaked with antidigoxigenin peroxidase for 30 minutes at RT. Tissues were stained with DAB and counterstained with hematoxylin. Slides then were cleaned, dehydrated, and stored. Apoptotic cells were thus detected by brown staining of the nuclei [63]. All experiments were performed in triplicate.

*(7) Evaluation of Labeling and Apoptotic Index*. The labeling index (LI) was determined by counting the proportion of Ki-67-positive nuclei per total number of cells. The apoptotic index (AI) was calculated by measuring the TUNNEL-positive cell percentage to the total cell number.

### 2.9. Statistical Analysis

The findings were set to mean ± standard mean error (SEM). Statistical assessment was carried out using the *t*-test and one-way variance analysis (ANOVA) using graph pad prism techniques, further verified by post hoc measurement check (Dunnett's *t*-test), and difference was found to be statistically significant by using *p* < 0.05. All assays have been conducted in triplicate.

## 3. Results

### 3.1. Pharmacophore Analysis

It is of interest to design the inhibitors for the breast cancer target synuclein gamma (SNCG) protein, using molecular docking-based virtual screening followed by molecular docking. The protein structures of SNCG were retained from PDB, and the resultant structures were used for molecular modeling methods to check the resolution of amino acid arrangement within the complex protein structures and predict the active site of the amino acids. The docking results shows that SNCG interacted significantly with ruthenium-fluvastatin within the active site amino acids of both polar and electrostatic charges within the target amino acids of Thr59, Asn64, Val66, Ser67, Glu68, and Val71 with substantial energy of -23.168 kcal/mol (Table 1 and Figures 1(b)–1(d)).

### 3.2. Instrumental Analysis

**Figure 2:**
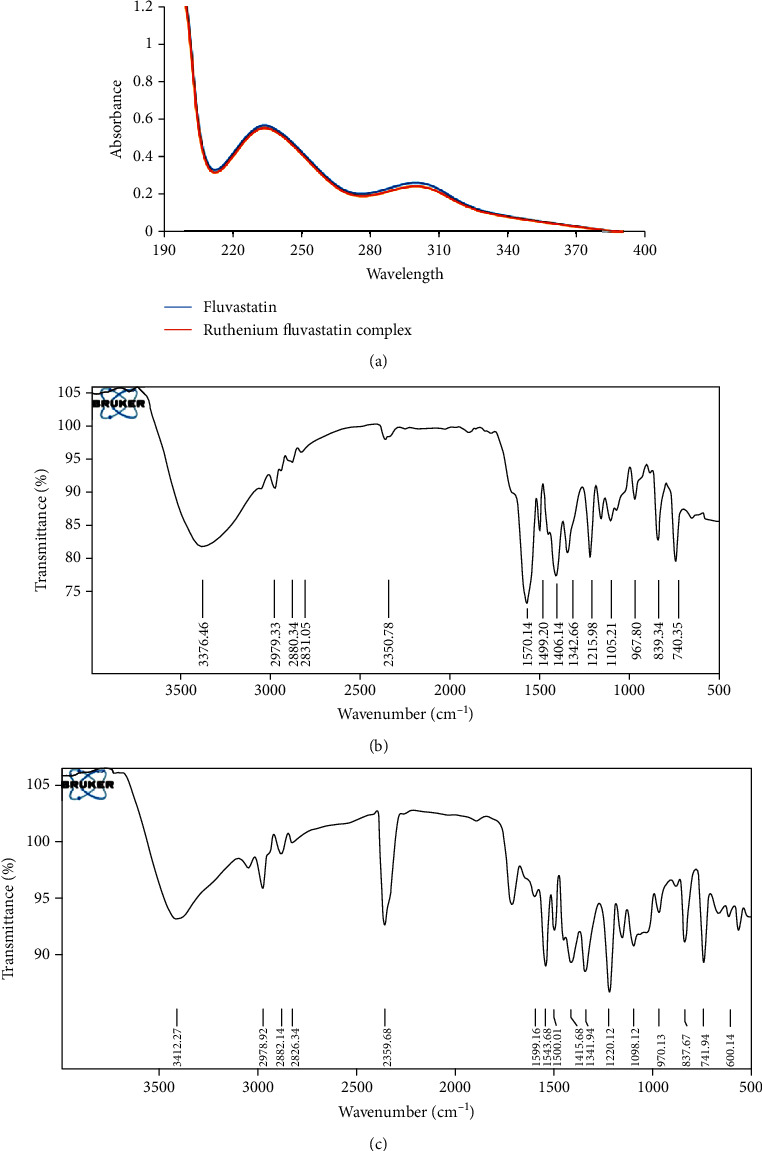
(a) UV-visible spectra of fluvastatin and the ruthenium-fluvastatin complex. (b) FTIR spectra of fluvastatin. (c) FTIR spectra of the ruthenium-fluvastatin complex.

**Figure 3:**
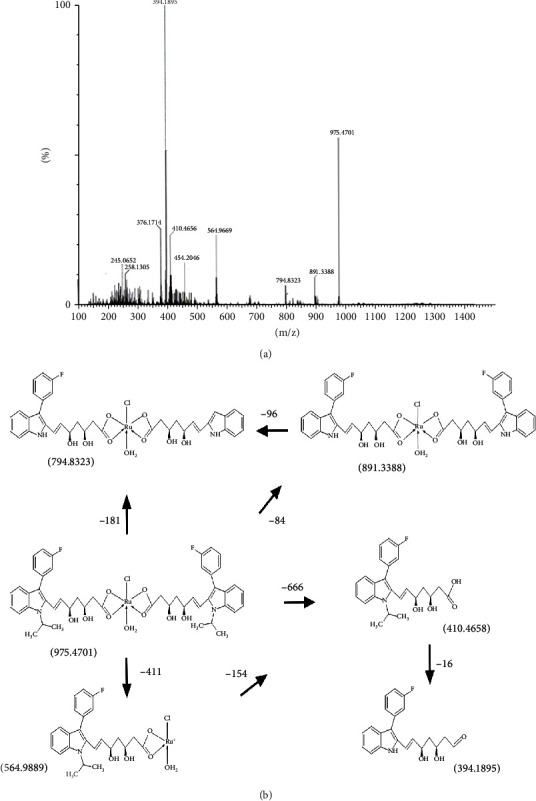
(a) Mass spectroscopy of the ruthenium-fluvastatin complex. (b) Possible fragmentation mechanism of the ruthenium-fluvastatin molecule.

**Figure 4:**
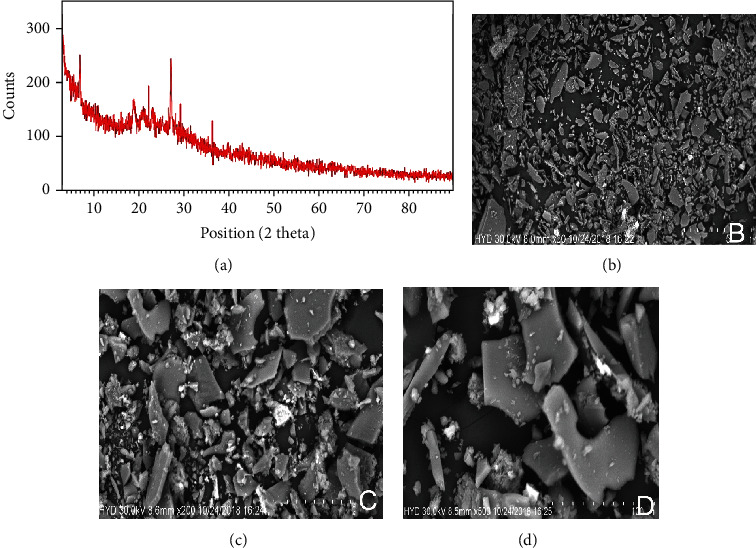
(a) X-ray diffractogram of the ruthenium-fluvastatin complex. Scanning electron microscopy (SEM) of the complex at (b) 200 *μ*m (c) 100 *μ*m, and (d) 50 *μ*m.

The electronic spectrum or UV spectrum of fluvastatin has shown absorption bands in two regions 230-240 nm and 300-310 nm due to interligand interaction. The complex only showed charge transfer between ligand to metal and metal to ligand as there was no appreciable change found in the UV-visible spectrum of the complex. So, no d-d transitions are expected due to the complex formation. The first range of the wavelength can be assigned to *π* → *π*∗ transitions in the aromaticity of the double bond. The other range of the wavelength is most probably due to the *n* → *π*∗ transition of mainly pyrrole, carboxylate, and hydroxyl group (Figure 2(a)). The FTIR study of the fluvastatin and ruthenium-fluvastatin complex was done to determine the coordination sites and binding properties of fluvastatin with ruthenium, as shown in Figures 2(b) and 2(c), and the analysis of the data was done in Table 2. The *ν* (O-H) frequencies appeared as broadbands at 3376.46 cm^−1^ in the case of fluvastatin, and in the case of the ruthenium-fluvastatin complex, the bands were found at 3412.27 cm^−1^ which shows the existence of water molecules. The *ν* (C-N) stretching mode in fluvastatin appeared at 1215.98 cm^−1^and was shifted to 1220.12 cm^−1^ after the complex formation. The *ν* (C-F) stretching appeared at 967.80 cm^−1^ in the spectrum of fluvastatin and shifted to 970.13 after the formation of the complex. At 1105.21 cm^−1^, a characteristic peak of *ν* (C-O) was found in fluvastatin and shifted to 1098.12 after complex formation. Another characteristic peak was found at 1570.14 cm^−1^ for *ν* (C=C) stretching, and that also shifted at 1543.68 cm^−1^ in the case of the complex. A characteristic peak for the formation of the metal oxide bond was found at 600.14 cm^−1^ (Figure 2(b)) which was absent in the case of the free fluvastatin. These results indicate that the oxygen group (=O and -O) is responsible for the chelation and formation of the ruthenium-fluvastatin complex. The ^1^H NMR spectrum of the complex shows that every signal or peak is also present in the spectrum of the complex with very less appreciable changes in their position as some sort of chemical shifts have been found after the formation of the complex; in the case of the complex, the appearance of signals of the protons of the two methyl groups slightly shifted at *δ* 1.350 ppm, two methylene groups of the 6-heptanoate as a multiplet shifted at *δ* 2.235-2.504 ppm, the three protons –CH-N and 2 –CH-OH as multiplet shifted at *δ* 4.996 ppm, and 2 –OH shifted at *δ* 4.754 ppm. The two protons of the double bond of the side chain give signal as doublet at *δ* 5.708 and 6.598 ppm, and the eight aromatic protons give signal as a multiplet at *δ* 7.004–7.445 ppm. Hence, it indicates that the chelation occurred on the carboxylate site. The mass spectrum of the complex is depicted in Figure 3(a) where the peak *m*/*z* at 975.47 designates the formation of the complex containing 2 fluvastatin+1 ruthenium+1 chloride+1 water molecule, the peak *m*/*z* 891.33 designates the 2 fragmented fluvastatin+1 ruthenium+1 chloride+1 water molecule, and the peak *m*/*z* at 410.46 shows the free fluvastatin. The various fragmentations of the complex are depicted in Figure 3(b). The surface morphology of the ruthenium-fluvastatin complex has been examined by SEM which is shown in Figures 4(b)–4(d); different magnifications of the sample were observed which correspond to the crystalline nature irregular form of the compound. The higher magnifications confirm the crystalline nature of the complex. The crystallinity as well as the physical state of the complex was further investigated by the X-ray diffraction study; Figure 4(a) shows the appearance of sharp peaks which are typical characteristics of crystalline nature of the complex; the sharp peaks were observed at different diffraction angles such as 7.09, 10.24, 19.05, 22.78, 27.27, 29.12, and 35.88A°. The sharp peaks in the diffractogram of the complex suggested that the complex is in crystalline nature.

### 3.3. *In Vitro* Antioxidant Capacity of the Ruthenium-Fluvastatin Complex

#### 3.3.1. Ruthenium-Fluvastatin Complex Was Capable of Scavenging DPPH, FRAP, and ABTS Radicals

**Figure 5:**
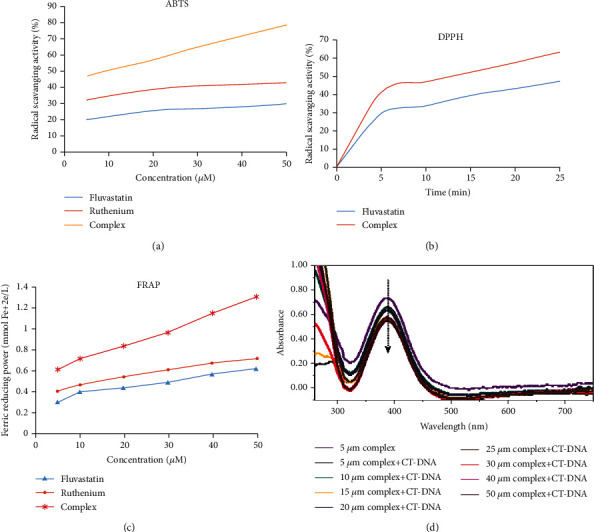
Measurement of antioxidant activity of the complex. Antioxidant activity of the ruthenium-fluvastatin complex by the (a) ABTS method, (b) DPPH method, and (c) FRAP method. (d) Absorbance spectra of CT-DNA in the presence of the ruthenium-fluvastatin complex.

Figure 5(a) indicates the scavenging actions of fluvastatin and ruthenium-fluvastatin by ABTS methods. Absorption of the effective ABTS solution at 734 nm was observed to substantially decrease in the presence of different concentrations of the complex. The complex's ABTS radical scavenging activity has been found to be better than that of the free fluvastatin. The presence of hydroxyl groups causes statins to have antioxidant activity. The complex's higher antioxidant activity is due to the hydroxyl groups, and their capacity to contribute hydrogen atoms improved after ruthenium chelation.

Figure 5(b) illustrates the different DPPH absorption patterns of fluvastatin and the ruthenium-fluvastatin complex at 515 nm, with increasing amount of time and concentration. With the increase in concentration and time, the complex showed higher DPPH absorption than the fluvastatin molecule. The complex represented better inhibitory effect compared to the free fluvastatin; the radical-sensitive Ru-O bond was introduced to the ruthenium-fluvastatin complex which synergistically enhances antioxidant activities of fluvastatin. In Figure 5(b), the plot illustrated the radical scavenging activity of fluvastatin and the complex, where it has been found that fluvastatin scavenged free radicals to about 47.10% and the complex scavenged about 63.17% at the same given reaction time.

The absorption of fluvastatin and the ruthenium-fluvastatin complex in the presence of Fe^+3^-TPTZ was estimated at 593 nm by absorbance variability during 10 min of FRAP reagent interaction with the subjected compound. The absorbance reduction is equivalent to the antioxidant quality.  Figure 5(c) shows that the antioxidant capacity of the complex is improved compared with free fluvastatin. These findings indicate that the complex of fluvastatin and ruthenium is capable of making a donation of protons and thus could have the ability to end a chain reaction. Metal chelation further increases the transfer of electrons from fluvastatin and thus augments the redox potential of the ruthenium-fluvastatin complex.

### 3.4. Ruthenium-Fluvastatin Complex Is Capable of Binding with CT-DNA

The broad absorption range in the presence of CT-DNA (5 microns) can be seen in Figure 5(d). A decline in the absorption rate (hypochromism) of the absorption peak is observed following the addition of the concentrations of the complex to CT-DNA. The intensity changes can be recognized within the intraligand transition band at 383 nm, after raising the complex concentration in the DNA. These absorption spectra disclose that the complex interacts with DNA by stacking action between the ligand's chromophore via the intercalative mode and the DNA base pairs.

### 3.5. *In Vitro* Assessment

#### 3.5.1. Ruthenium-Fluvastatin Complex Instigates the Repression of Cell Viability

**Figure 6:**
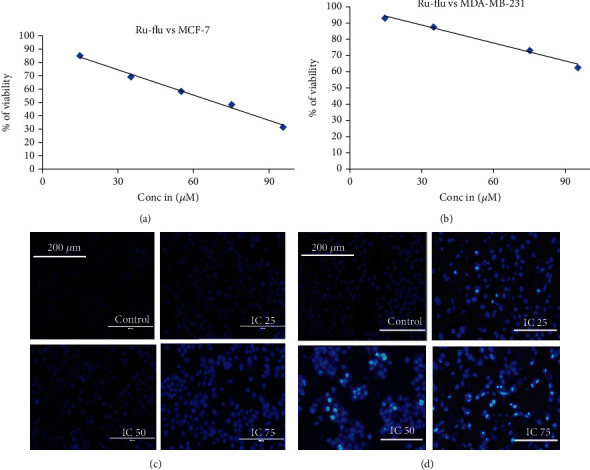
Effects of the ruthenium-fluvastatin complex on cell viability of (a) MCF-7 cells and (b) MDA-MB-231 cells at 24 hours. (c) DAPI-stained MCF-7 cells after 24 hours of treatment with the ruthenium-fluvastatin complex. (d) DAPI-stained MDA-MB-231 cells after 24 hours of treatment with the ruthenium-fluvastatin complex.

The MTT assay was performed to investigate the inhibitory effect of the ruthenium-fluvastatin complex on MCF-7 and MDA-MB-231 cells. The cell viability evaluation revealed that the ruthenium-fluvastatin complex showed a dose-dependent inhibitory impact on human breast cancer cells MCF-7 and MDA-MB-231 (Figures 6(a) and 6(b)). Treatment with the complex was observed to decrease the viability of the MCF-7 cells to 85%, 69%, 58%, 49%, and 31%, respectively, at doses of 15, 35, 55, 75, and 95 *μ*M. Likewise, findings were observed in MDA-MB-231 cells where cell viability decreased to 93%, 87%, 80%, 73%, and 62% at concentrations 15, 35, 55, 75, and 95 *μ*M after 24 hours. Exposure to the complex at 95 *μ*M in MCF7 cells was observed to have a maximum inhibition rate of 69%, while stimulation with 95 *μ*M of the complex on MDA-MB-231 cells displayed a maximum inhibition rate of 38% at 24 hours.

#### 3.5.2. Ruthenium-Fluvastatin Complex Causes Chromatin Condensation

Cells containing condensed chromatin morphologically usually signify that the cells might be undergoing apoptosis and fluorescence with bright blue color in the presence of DAPI. Treatment with the complex causes dose-dependent nuclear condensation in both cell lines (Figures 6(c) and 6(d)). It has been observed that intervention with 100 *μ*M of the complex in MCF-7 cells and 200 *μ*M of the complex in MDA-MB-231 cells displayed maximum chromatin condensation after 24 hours.

#### 3.5.3. Ruthenium-Fluvastatin Complex Encourages Colony Inhibition Capability

**Figure 7:**
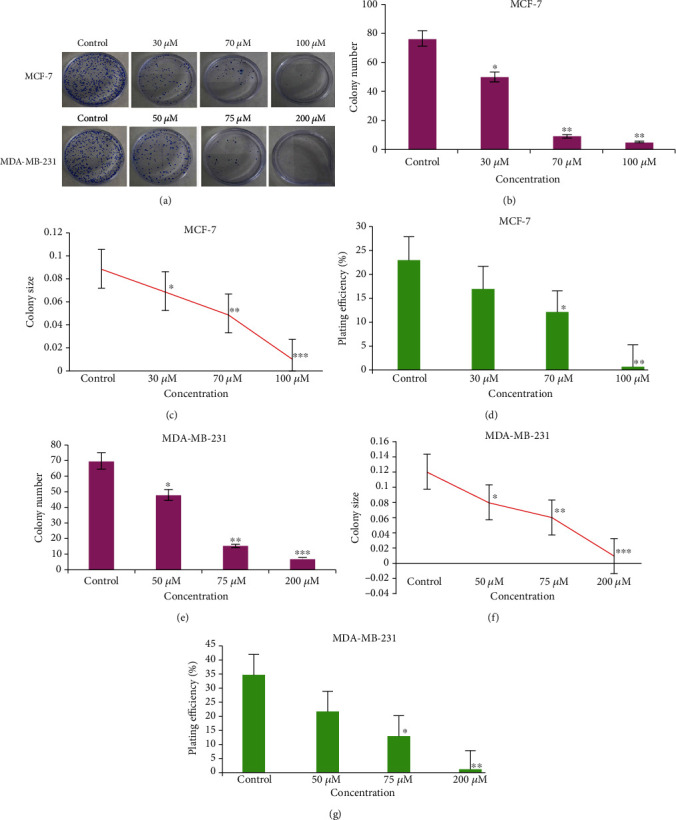
(a) Clonogenic assay of MCF-7 and MDA-MB-231 cells after 24 hours of treatment with the ruthenium-fluvastatin complex. (b) Quantification of the colony number for MCF-7 cells. (c) Quantification of colony size of the clonogenic assay of MCF-7 cells. (d) Plating efficiency for the clonogenic assay of MCF-7 cells. (e) Quantification of the colony number for MDA-MB-231 cells. (f) Quantification of colony size for MDA-MB-231 cells. (g) Plating efficiency for the clonogenic assay of MDA-MB-231 cells. Quantification of the colony number and size was performed using the ImageJ public domain software. Data represent means ± SD from three different experiments in triplicate. The results were compared using ANOVA, followed by Tukey's post hoc analysis. Asterisks represent ^∗^*p* < 0.05, ^∗∗^*p* < 0.01, and ^∗∗∗^*p* < 0.001 as compared to carcinogen control.

The relevance of the tumor-colony forming assessment for screening new drugs has often been identified as an important tool for research. The ruthenium-fluvastatin complex effectively stimulates the capacity of MCF-7 and MDA-MB-231 cells to inhibit colony formation (Figure 7(a)). In MCF-7 and MDA-MB-231 cells, the ruthenium-fluvastatin complex was substantially successful in suppressing the colony number (Figures 7(b) and 7(e)) and size (Figures 7(c) and 7(f)) relative to control cells. Plating efficiency (PE) was measured for the ruthenium-fluvastatin complex for both MCF-7 and MDA-MB-231 cells (Figures 7(d) and 7(g)), and the findings indicate that PE was substantially decreased in MCF-7 and MDA-MB-231 at the maximum doses of the ruthenium-fluvastatin complex.

#### 3.5.4. Ruthenium-Fluvastatin Complex Initiates Apoptosis Arrests of the Cell Cycle

**Figure 8:**
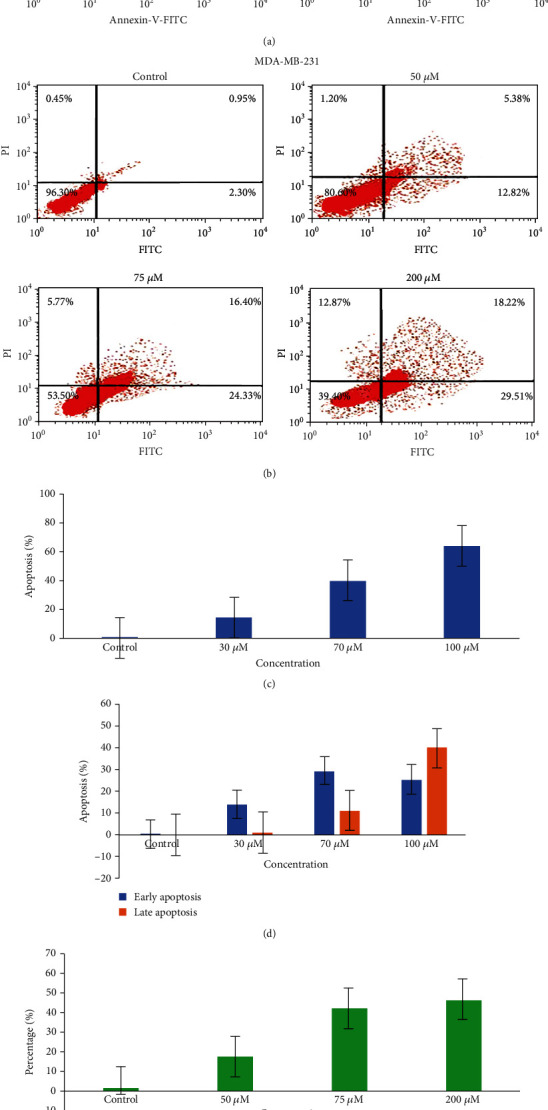
(a) Detection of apoptosis in MCF-7 cells by flow cytometry after treatment with the ruthenium-fluvastatin complex. (b) Detection of apoptosis in MDA-MB-231 cells by flow cytometry after treatment with the ruthenium-fluvastatin complex. (c) Percentage of apoptotic cells versus concentration in MCF-7 cells. (d) Percentage of apoptotic cells in the early and late apoptosis stage in MCF-7 cells. (e) Percentage of apoptotic cells versus concentration in MDA-MB-231 cells. (f) Percentage of apoptotic cells in the early and late apoptosis stage in MDA-MB-231 cells.

MCF-7 and MDA-MB-231 undergoing apoptosis were observed by staining them with annexin V and PI by treating them with three different concentrations, respectively, of the complex for 24 hours. A flow cytometric study can differentiate stained cells into four categories, namely, viable (annexin V-PI-), early apoptosis (annexin V+PI-), late apoptosis (annexin V+PI+), and necrotic (annexin V-PI+) cells. Figures 8(a) and 8(b) show the division of cells receiving therapy with specific complex concentrations after 24 hours. The percentages of apoptotic cells are 14.31, 39.91, and 64.25% after exposure to 30, 70, and 100 *μ*M of the complex in MCF-7 cells and 18.2, 40.73, and 47.73% following treatment with 50, 75, and 200 *μ*M of the complex in MDA-MB-231 cells as compared to control (Figures 8(c) and 8(e)). Besides, a dose-dependent increase was also identified in the early apoptotic cells after 24 hours of complex therapy (Figures 8(d) and 8(f)).

**Figure 9:**
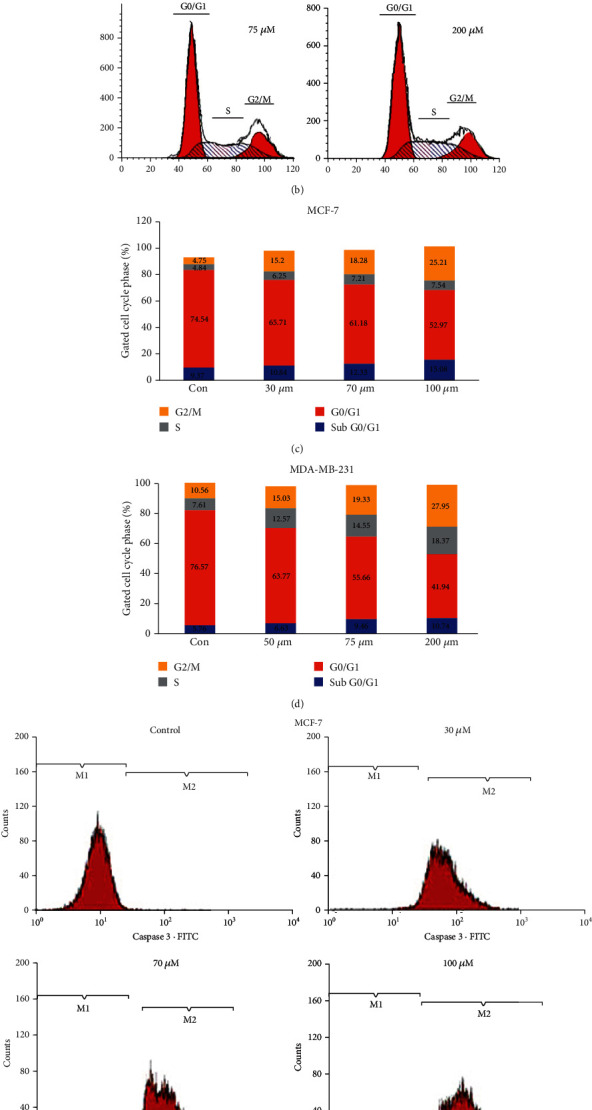
(a) Analysis of cell cycle phase distribution of MCF-7 cells after the treatment with the ruthenium-fluvastatin complex. (b) Analysis of cell cycle phase distribution of MDA-MB-231cells after the treatment with the ruthenium-fluvastatin complex. (c) Quantitative distribution of MCF-7 cells in different phases of the cell cycle. (d) Quantitative distribution of MDA-MB-231 cells in different phases of the cell cycle. (e) Expression of caspase-3 proteins in MCF-7 cells after 24 hours of treatment with the ruthenium-fluvastatin complex. (f) Expression of caspase-3 proteins in MDA-MB-231 cells after 24 hours of treatment with the ruthenium-fluvastatin complex.

Flow cytometric assessment was used to analyze the proliferation processes of the cell cycle along with the content of the cellular DNA. The proportion of subdiploid cells within the cell cycle histogram is the indication of the apoptotic cells (Figures 9(a) and 9(b)). MCF-7 cells exposed to 30 *μ*M, 70 *μ*M, and 100 *μ*M complex concentrations indicated 65.71%, 61.18%, and 52.97% cells in the G0/G1 stage. Comparable findings were reported in MDA-MB-231 cells, where exposure to 50 *μ*M, 75 *μ*M, and 200 *μ*M of the complex yielded 63.77%, 55.66%, and 41.94% of cells in the G0/G1 stage. At the same time, a rise in cells in the S phase was observed for both cell types in a dose-dependent fashion after complex therapy (Figures 9(c) and 9(d)). It should also be reported that the complex causes a dose-dependent decline in the number cells in the G0/G1 stage for both cell lines.

#### 3.5.5. Ruthenium-Fluvastatin Complex Initiates Caspase-3-Mediated Apoptosis

Quantification of caspase-3 was performed in triplicate in MCF and MDA-MB-231cells, by using flow cytometric analysis, and the results have been determined depending on the percentage of the cell population in respect to the caspase-3 enzymatic activity throughout the apoptotic process. The flow cytometric evaluation of the effect of caspase-3 ruthenium-fluvastatin complex treatments on both cell types after 24 hours is shown in Figures 9(e) and 9(f). The quadrant M1 represented the number of live cells without caspase-3, while quadrant M2 corresponds to the number of active caspase-3-designated apoptotic cells. After drug therapy, the percentage of caspase-3-demarcated apoptotic cells in quadrant M2 is substantially better than that of viable cells in quadrant M1, while unexposed cells display a lower percentage of viable cells in quadrant M1 relative to the caspase-3-demarcated apoptotic cells in quadrant M2. This observation is at par with the result of the apoptotic analysis, as an increment of the caspase-3 corresponds with the increase in the late apoptotic cells.

#### 3.5.6. Ruthenium-Fluvastatin Complex Modulates Expression of PI3K, Akt, mTOR, EGFR, VEGF, and Cleaved Caspase-3

**Figure 10:**
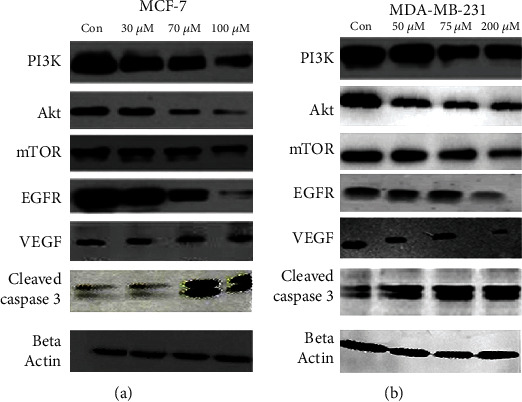
(a) Western blot analysis of expressions of PI3K, Akt, mTOR, EGFR, VEGF, and cleaved caspase-3 in MCF-7 cells. (b) Western blot analysis of expressions of PI3K, Akt, mTOR, EGFR, VEGF, and cleaved caspase-3 in MDA-MB-231 cells.

Western blot experimentation was performed to establish the inhibition of MCF-7 and MDA-MB-231 cell growth by the complex through diverse cell cycle modulatory factor variation. We verified the effects of ruthenium-fluvastatin complex treatment on various proteins like PI3K, Akt, mTOR, EGFR, VEGF, and cleaved caspase-3 in MCF-7 and MDA-MB-231 human breast cancer cells. After 24 hours of exposure to the ruthenium-fluvastatin complex in both MCF-7 and MDA-MB-231 cells, a dose-dependent downregulation of PI3K, Akt, mTOR, EGFR, and VEGF was identified (Figures 10(a) and 10(b)). Nonetheless, a significant upregulation of cleaved caspase-3 was observed in both MCF-7 and MDA-MB-231 cells after 24 hours of exposure to ruthenium-fluvastatin therapy.

### 3.6. Toxicity Study

#### 3.6.1. Acute and Subacute Toxicity Study

The LD_50_ dosage of the ruthenium-fluvastatin complex was estimated to be 300 mg/kg. 25, 50, 100, and 200 mg/kg were chosen as the subacute toxic doses following the LD_50_ dose evaluation. No treatment-related deaths in animals treated with 25, 50, 100, or 200 mg/kg of the complex were recorded during subacute toxicity evaluation (28 days).

### 3.7. Analysis of Hematological and Serum Biochemical Parameters

Tables 3–6 demonstrate the serum biochemical and hematology analysis of the treated and control animals. WBC and RBC quantities in the ruthenium-fluvastatin complex (200 mg/kg) dose groups were substantially improved in comparison to those of control animals. ALP, ALT, and AST were slightly higher than those of the control group at 200 mg/kg dose (*p* < 0.05). Glucose and BUN were both changed (*p* < 0.05) in animals administered with 200 mg/kg of drug. The complex at a dose of 200 mg/kg induces toxicity in animal models to some extent and was thus not considered a standard for subsequent research.

### 3.8. Histopathology

**Figure 11:**
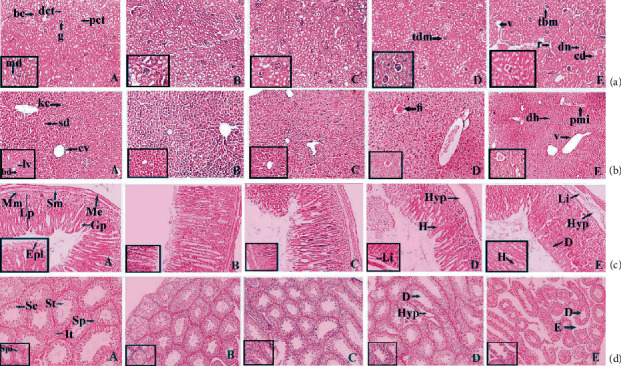
(a) Histopathological representation of the kidney of Balb/c mice: (A) normal tissue showing the glomerulus (g), Bowman's capsule (bc), macula densa (md), proximal convoluted tubule (pct), and distal convoluted tubule (dct); (B) kidney tissue exposed to 25 mg/kg complex; (C) kidney tissue exposed to 50 mg/kg complex; and (D) kidney tissue exposed to 100 mg/kg complex and (E) exposed to 200 mg/kg complex showing thickening of the capsular membrane (tbm), ruptures (r), desquamated nuclei (dn), and vacuolization. (b) Histopathological representation of the liver of Balb/c mice: (a) normal control showing the central vein (cv), bile duct (bd), sinusoidal dilation (sd), Kupffer cell (kc), and lymph vessel (lv); (B and C) kidney tissue exposed to 25 mg/kg and 50 mg/kg of the complex, respectively; and (D and E) kidney tissue exposed to 100 and 200 mg/kg complex showing the periportal mononuclear infiltrates (pmi), degeneration of hepatocytes (dh), and focal inflammation (fi). (c) Histopathological representation of the stomach of Balb/c mice: (a) normal control showing the muscularis externa (Me), submucosa (Sm), muscularis mucosa (Mm), lamina propria (Lp), gastric pit (Gp), and epithelial lining (Epl); (B and C) stomach tissue exposed to 25 mg/kg and 50 mg/kg of the complex, respectively; and (D and E) stomach tissue exposed to 100 and 200 mg/kg complex showing hemorrhages (H) between the villus, hyperplasia (Hyp), and leukocyte infiltration (Li). (d) Histopathological representation of the testis of Balb/c mice: (a) normal control showing the Sertoli cell (Sc), spermatogonia (Sp), seminiferous tubule (St), and interstitial tissues (It) seen within the tubular lumen; (B and C) testis exposed to 25 mg/kg and 50 mg/kg of the complex, respectively; and (D and E) testis exposed to 100 and 200 mg/kg complex showing edema in interstitial tissue (E), degeneration of the seminiferous tubule (D), and hyperplasia (Hyp). H&E: 10x magnification (inset: 40x).

Histopathology of the kidney (Figure 11(a) A) of the control group exhibited the normal arrangement of architectural organization. The principal morphological disparity was observed at the dose of 200 mg/kg (Figure 11(a) E). 25 and 50 mg/kg doses did not suggest any significant animal anomalies (Figure 11(a) B and C), while mild Bowman's capsule thickening was found in animals administered with 100 mg/kg complex doses (Figure 11(a) D). Capsular membrane thickening (tm), cytoplasmic debris (cd), pyknotic nucleus (pn), vacuolization (v), and node sclerosis (n) were detected at 200 mg/kg doses of the complex. Liver histopathology (Figure 11(b) A) denoted normal hepatic structures in the control population while maximal doses (200 mg/kg) of the ruthenium- fluvastatin complex delineated focal inflammation (fi), hepatocyte degeneration (d), and mononuclear periportal infiltration (pmi) (Figure 11(b) E). Animals given doses of 25, 50, and 100 mg/kg showed no critical deformity (Figure 11(b) B–D). Figure 11(c) A shows the microscopic examination of the stomach, where a dosage of 200 mg/kg of the ruthenium-fluvastatin complex revealed congestion (c), hemorrhages (H), and hyperplasia of the glandular gastric zone (Hyp) (Figure 11(c) E). Yet histopathological differences were not found at the lower dose range (25 mg/kg, 50 mg/kg, and 100 mg/kg) (Figure 11(c) B–D). Figure 11(d) A shows the microscopic evaluation of tests where 200 mg/kg (Figure 11(d) E) of the ruthenium-fluvastatin complex administered in the animal population displayed degeneration in seminiferous tubules (D) and edema in interstitial tissues (E), and at 100 mg/kg dose level (Figure 11(d) D), degeneration (D) and hyperplasia (Hyp) were detected, but no histopathological changes were found at the lower dosage points (25 mg/kg and 50 mg/kg) (Figure 11(d) B and C).

### 3.9. *In Vivo* Carcinogenesis Study

#### 3.9.1. Histopathology of Mammary Tissue

**Figure 12:**
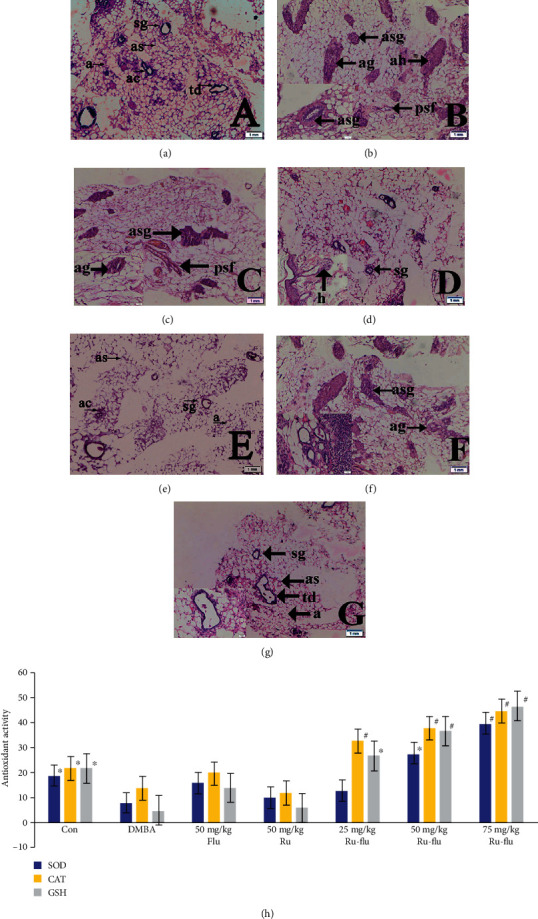
(a) Histological appearance of mammary tissue of normal control showing the terminal duct lobular units (td), alveoli (a), alveolar septa (sg), acinus (ac), and serous gland (sg). (b) DMBA control shows atrophy of glands with periductal stromal fibrosis and fatty tissue (psf), atrophy of glands (ag) with surrounding fatty tissue, atrophy of serous glands (asg) with surrounding stromal fibrosis, and atypical hyperplasia (ah). (c) Mammary tissue of the DMBA-induced group treated with 25 mg/kg ruthenium-fluvastatin complex showing atrophy of serous glands (asg), atrophy of glands (ag), and periductal stromal fibrosis and fatty tissue (psf). (d) Mammary tissue of the DMBA-induced group treated with 50 mg/kg ruthenium-fluvastatin complex showing hyperplasia of serous and mucinous glands (h). (e) Mammary tissue of the DMBA-induced group treated with 75 mg/kg ruthenium-fluvastatin complex having almost normal architecture. (f) Mammary tissue of the DMBA-induced group treated with 50 mg/kg ruthenium. (g) Mammary tissue of the DMBA-induced group treated with 50 mg/kg fluvastatin. (h) Effect of the ruthenium-fluvastatin complex on in vivo antioxidant enzymes: SOD (superoxide dismutase), CAT (catalase), and GST (glutathione). ^∗^*p* < 0.05 as compared to the carcinogen control; ^#^*p* < 0.01 as compared to the ruthenium, fluvastatin, and ruthenium-fluvastatin (25, 50, and 100 mg/kg).

The normal control (group I) illustrates normal alveolar septa (as), alveoli (a), acinus (ac), serous gland (sg), and terminal duct lobular units (td) of mammary tissue kept intact as seen in Figure 12(a). The DMBA-treated section of group II animals revealed atrophy of the periductal, stromal, and fatty tissue glands (psf), atrophy of the underlying fatty tissue (ag), atrophy of the serous glands (asg) surrounding stromal fibrosis, and hyperplasia of the serous and mucous glands (ah) in their mammary tissues (Figure 12(b)). Slight hyperplasia of serous and mucinous glands (Figures 12(c) and 12(d)) was seen in the histological analysis of 25 and 50 mg/kg ruthenium-fluvastatin complex-treated groups while in the highest dose category (100 mg/kg), there has been no evidence of hyperplasia or cell proliferation in mammary tissue and normal morphology of the cells covering the ducts was observed (Figure 12(e)). The 50 mg/kg fluvastatin-treated model revealed typical histological composition of rat mammary tissue (Figure 12(g)), while 50 mg/kg ruthenium-treated animals showed gland atrophy with surrounding fatty tissue (ag) and serous gland atrophy (asg) (Figure 12(f)).

#### 3.9.2. Antioxidant Evaluation of Mammary Tissues

The fragmented mammary tissue of the carcinogen control animals has been observed with reduced levels of SOD and CAT and reduced glutathione. The animals treated with 100 mg/kg ruthenium-fluvastatin complex reported a marked rise in SOD, CAT, and glutathione quantities in the homogenized mammary tissues as compared to carcinogenic control and other groups (Figure 12(h)).

#### 3.9.3. Immunohistochemical Evaluation of Mammary Tissues

**Figure 13:**
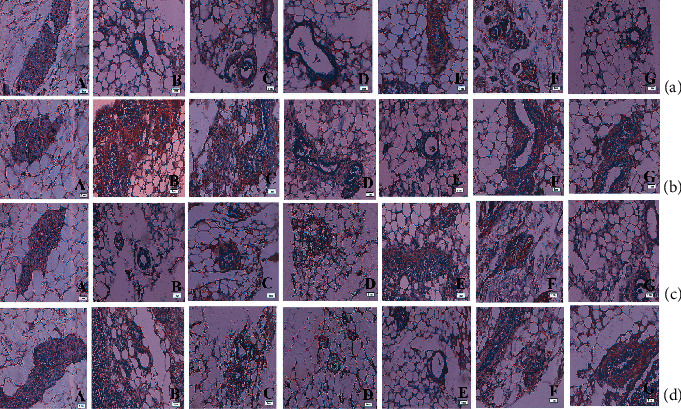
The immunohistochemical analysis of the (a) p53, (b) Bcl2, (c) Bax, and (d) MMP9 expressions in the mammary tissues of different groups of rats: (A) the normal control, (B) carcinogen control, (C) 25 mg/kg complex-treated rats, (D and E) 50 and 100 mg/kg complex-treated rats, (F) 50 mg/kg fluvastatin-treated animals, and (G) 50 mg/kg ruthenium-treated animals. All images at 40x.

To outline the influence of ruthenium-fluvastatin therapy on mammary cancer in rats, immunohistochemical staining approaches have been used to determine the existence of cellular biomarkers such as Bax, Bcl2, p53, and MMP9 (Figure 13) (Table 7). It was found that DMBA treatment greatly raised levels of Bcl2 (Figure 13(b) B) and MMP9 (Figure 13(d) B), while also downregulating the levels of Bax (Figure 13(c) B) and p53 (Figure 13(a) B) as compared to the control group (A in Figures 13(a)–13(d)) (*p* < 0.05). The expression of Bax (Figure 13(c) C–E) and p53 (Figure 13(a) C–E) was greatly improved by ruthenium-fluvastatin therapy, but Bcl2 (Figure 13(b) C–E) and MMP9 (Figure 13(d) C–E) were significantly decreased after ruthenium-fluvastatin therapy. The dosage of 100 mg/kg ruthenium-fluvastatin complex was effective in raising the concentrations of Bax and p53 while the concentrations of Bcl2 and MMP9 (*p* < 0.01) showed a substantial decrease relative to those of carcinogen-treated animals. The presence of the above-mentioned biomarkers allows one to believe that the complex focuses on apoptosis and thus regulates the cell cycle to effectively constrain the progression of the disease.

#### 3.9.4. Inhibition of Ki-67 by the Ruthenium-Fluvastatin Complex in the Mammary Tissue

**Figure 14:**
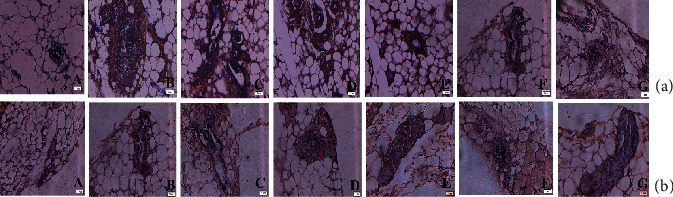
(a) The immunohistochemical analysis of expression of Ki-67 of different groups of rats: (A) the normal control, (B) carcinogen control, (C) 25 mg/kg complex-treated rats, (D and E) 50 and 100 mg/kg complex-treated rats, (F) 50 mg/kg fluvastatin-treated animals, and (G) 50 mg/kg ruthenium-treated animals. All images at 40x. (b) TUNEL assay of apoptotic cells: (A) the normal control, (B) carcinogen control, (C) 25 mg/kg complex-treated rats, (D and E) 50 and 100 mg/kg complex-treated rats, (F) 50 mg/kg fluvastatin-treated animals, and (G) 50 mg/kg ruthenium-treated animals. All images at 40x.

The potency of the ruthenium-fluvastatin molecule in mammary tissue proliferation has been depicted in Figure 14(a). The LI (labeling index) is measured as a proportion of Ki-67 tagged cells shown in Table 8. A substantial improvement in the Ki-67-LI activity was found in the DMBA-treated animals (Figure 14(a) B) compared with the normal control group (Figure 14(a) A), but a small decrease in the Ki-67-LI value was found in the highest dose of ruthenium-fluvastatin complex-treated animals (*p* < 0.01) (Figure 14(a) C–E) relative to carcinogen control animals.

#### 3.9.5. Ruthenium-Fluvastatin Complex Promotes Apoptosis in Mammary Tissue

The TUNNEL assay was carried out to assess the outcome of apoptosis with ruthenium-fluvastatin therapy in mammary carcinogenesis (Figure 14(b)). Apoptosis prompts the nuclear DNA to be fragmented into different segments that create DNA strand breaks that could be identified by the brown marks produced by DAB chromogen. Normal control cells undergoing cell death have been displayed in (Figure 14(b) A). In the carcinogen control group, the TUNEL-labeled cells enduring apoptosis were very limited (Figure 14(b) B), while the TUNEL-labeled cells of ruthenium-fluvastatin-treated animals dramatically increased (Figure 14(b) C–E). Typically, 3 to 5 apoptotic cells were found in an environment of approximately 700 cells throughout the carcinogen control group, which increased to 10-14 cells per 700 cells in the 100 mg/kg of the complex-administered animals. AI specifies the apoptotic index and appears in Table 8. Animals obtaining 100 mg/kg of the drug, when compared with the carcinogen control group, represented a marked increase in apoptosis. The *R* value represents the relationship between cell proliferation and apoptosis. Cell proliferation and TUNEL evaluation suggest that the new improvements in the tumor's microenvironment could be followed by a parallel rise in cell proliferation and a minimization of cell death. The *R* value hits a plateau in the carcinogen control group; however, it gradually decreases with the complex's increasing concentration. Through mentioning both of these hypotheses, we can conclude that the complex activates apoptosis and ultimately decreases cell proliferation in a dose-dependent manner.

## 4. Discussion

Current anticancer drugs obtained from metal complexes focus entirely on activating cell apoptosis and offer substantial improvements in pharmacological studies [64]. This change was spectacularly motivated by the discovery of platinum-based antitumor drugs, but various obstacles such as intense side effects, drug resistance, mutation aggregation, and epimutations cause to come up with substitute therapies. In their research paper, Allardyce and Dyson explained that another platinum group metal, ruthenium, exhibits similar propitious biological properties [65] and is further able to establish strong chemical bonds through variable electronegativity thus rendering it capable to interact with a variety of biomolecules [66].

A lot of emerging chemotherapeutics utilize apoptosis as a mechanism to induce cellular death in tumor cells [67]. To prevent apoptosis, a tumor may gain a variety of mutations or alterations. While escaping from programmed cell death is a crucial feature of tumorigenesis, it does not appear to be a common response to all apoptotic stressors. Arguably, some of the apoptotic mechanisms and pathways in tumors stay unchanged, making them ideal candidates for therapeutic targeting [68]. Among other molecules, statins have been found to have apoptosis-inducing properties. The potency of statins as an anticancer therapy has been explored both in monotherapy and in combined regimens with commonly used chemotherapeutics [69]. Several reports have also indicated that statins caused programmed cell death in a subset of tumor-derived cell lines in vitro, indicating that the analogous cancers might be susceptible to statin-specific apoptosis in vivo [70–72]. Thus, the present study focuses on exploring the potential effects of the ruthenium-fluvastatin complex on in vitro and in vivo breast cancer models.

Synuclein-*γ* (SNCG) is a member of the synuclein family that has been associated with both neurodegenerative disorder and cancer [73]. A collection of functional experiments has shown that SNCG's ectopic expression in breast carcinoma facilitates proliferation and migration [34]. SNGC inhibitors have been investigated extensively based on previous studies and offer significant probability as a future drug target [74]. Inspired by this data and extensively exploring this problem, we decided to take benefit of molecular docking analyses to evaluate and explore the novel complex's binding mechanism against SNCG as a target protein.

Our findings showed that the unrestricted binding energy for the complex was low, thus promoting the binding direction of the compounds in the SNCG binding pocket circling the active site, resulting in enzyme inhibition. Since SNCG is one of the prominent markers in breast carcinoma, our binding studies denote that the novel complex could also modulate the SNCG proteins in breast carcinoma. In fact, our research included the synthesis and characterization of the complex. We utilized different spectroscopic evaluations to evaluate fluvastatin's antioxidant capacity pre- and postcomplex formation. Results confirm that the oxygen group (=O) and hydroxyl group are responsible for the chelation and formation of the ruthenium-fluvastatin complex and that it is crystalline in nature. The analysis of antioxidant activity showed that the mechanism of free radical scavenging of fluvastatin on resultant metal complexation is greatly enhanced. Ruthenium thus promotes the modification of fluvastatin's oxidative ability after complexation by enhancing the transfer of electrons from fluvastatin and thus raising its redox potential. The complex's reaction towards CT-DNA led to a reduction in the absorption spectra as compared to that of uncombined DNA, thus confirming that the complex binds through the intercalation mode with CT-DNA.

The next research section was devoted to evaluating the impact of the ruthenium-fluvastatin complex on the cancer cell lines MCF-7 and MDA-MB-231. The MTT assay showed that the ruthenium-fluvastatin complex can minimize cellular proliferation and promote apoptosis. One of major significant aspects of anticancer therapies is the modulation of the cell cycle; specifically, the inhibition of phases G1 and G2 plays a key role in the cell cycle cascade [75]. To determine the complex's mechanistic approach to the induction of apoptosis, flow cytometric experiments were employed that made use of annexin V and PI staining procedures. Furthermore, the results revealed that a higher percentage of early apoptotic events were identified on both MCF-7 and MDA-MB-231 cancer cells by ruthenium-fluvastatin treatment, resulting in the arrest of the G0/G1 point, amounting to cellular death.

In addition, a cell-oriented reporter analysis was performed to identify the effects of complex treatment on the presence of PI3K-, Akt-, mTOR-, EGFR-, and VEGF-associated signaling trails. The PI3K/Akt/mTOR pathway is a cell signaling cascade associated with growth modulation, proliferation, reproduction, motility, metabolism, and immune response [76, 77]. The mammalian target of rapamycin (mTOR) strongly engages in various tumor progression processes by activating the signaling pathway PI3K/Akt [78]. Alterations are found in nearly all human tumors, especially with breast cancer, of which up to 60% of tumors represent unique configurations that activate this cascade [79]. Dysregulation of this mechanism covers a wide variety of cancer symptoms including unchecked proliferation, genomic disturbance, and metabolic reconfiguring in tumor cells [64, 80]. In fact, activation of the PI3K/Akt/mTOR pathway is one of the major causes of current cancer chemotherapy resistance [81]. It is aimed at making the PI3K/Akt/mTOR pathway a critical research target for understanding the development and progression of this disease, and the importance of this pathway as a potential therapeutic approach along with the prognostic and diagnostic value of this pathway in patients with breast cancer is undeniable [82, 83]. Thus, our studies denote that the ruthenium-fluvastatin complex significantly downregulates PI3K, Akt, and mTOR in both MCF-7 and MDA-MB-231 cells.

Apart from these, the growth factor of the epidermis and its receptors (EGFR) in breast cancer are continuously often overexpressed [84]. EGFR is a transmembrane tyrosine kinase receptor that regulates cell proliferation and epithelial cell viability via the PI3K/Akt/mTOR and protein kinase mitogen-activated (MAPK) signaling cascade [85]. The epidermal growth factor pathway serves as a primary mediator for breast cancer initiation and progression by encouraging the proliferation of cancer cells and their survival and promoting resistance to conventional therapy [86]. In cancer chemotherapeutics, epidermal growth factor receptors and their ligands are thoroughly studied due to their mutation and overexpression in a large segment of primary breast carcinomas [87–89]. Likewise, the growth regulation signaling cascade involving the vascular endothelial growth factor (VEGF) binds to the VEGF receptor 2 (VEGFR2) and activates tumor vasculature [90]. Therefore, the inhibition of the signaling pathways EGFR and VEGF is also a promising technique for cancer chemotherapy [91]. Our western blot findings provide definitive proof that the complex operates in both MCF-7 and MDA-MB-231 cells via the EGFR and VEGF pathways by downregulation of their signals.

Apoptosis is a crucial and essential method of ideally programmed cell death that involves eliminating dysfunctional cells in mammalian development and retaining tissue homeostasis. Apoptotic activation has been considered an essential and effective cancer treatment approach [92]. In our current research, the DAPI staining approach used fluorescence microscopy in MCF7 and MDA-MB231 cells to explore modification of nuclear morphology. Treatment with the ruthenium-fluvastatin complex has shown condensed chromatin and scattered nuclei, which specifically demonstrate apoptosis induction in these cells. In breast cancer, in vitro studies have shown that dysregulated caspase activity is involved in chemotherapeutic resistance. One study demonstrated that restoration of caspase-3 expression, in caspase-3-deficient MCF-7 breast cancer cells, can sensitize to doxorubicin and etoposide-induced apoptosis, suggesting caspase-3 deficiency may be a possible mechanism for chemoresistance [93, 94]. Our study is at par with previous literature denoting an increase in the caspase-3 activity after drug treatment indicating that the complex is capable of modulating the caspase-3 expression owing to the increased amount of apoptotic events.

The global harmonized program for categorizing and marking chemicals involves the reporting of a safe dose for a novel anticancer molecule [95]. Therefore, an acute and subacute toxicological analysis was performed to determine the LD_50_ value and appropriate doses of the complex. The findings of our in vivo work further indicate that the ruthenium-fluvastatin complex functions through the escalation of the proteins caspase-3 and p53 and also downregulates the expression of PI3K, Akt, mTOR, EGFR, and VEGF. Recent research is reportedly centered on the role of p53 in regulating the growth of cells induced by intense oncogenic signals or replicative stress [96]. p53 controls the function of large numbers of target genes related to cell cycle capture, DNA repair, senescence, and apoptosis when activated [97]. Contrary to other cells, cancer cells exhibit elevated mutation levels in the p53 gene. p53-dependent p21 upregulation induces misregulation of DNA replication and has been documented in active cancer cells [98]. Upregulation of p53 in tumors has been documented to cause senescence-induced tumor progression [99]. In addition, the activation of the prosurvival and antiapoptotic proteins causes the cancer cells to proliferate and survive. This mechanism facilitates tumor development and the progression of the disease. DNA damage modulates p53-based signals from a molecular point of view, which also contributes to proapoptotic stimuli [100, 101]. Proapoptotic proteins such as Bax disrupt the mitochondrial membranes and promote the release of cytochrome c and other proapoptotic stimuli through the use of antiapoptotic proteins such as Bcl2 and BclxL [102]. Our western blot and immunohistochemical results revealed that expression of p53, caspase-3, and Bax was upregulated, while the role of Bcl2 proteins was downregulated, thus endorsing our hypothesis that the novel complex operates via the intrinsic apoptotic pathway Bax and Bcl2 supported by p53.

Consequently, MMP9, which belongs to a class of zinc-dependent endopeptidases, was downregulated with ruthenium-fluvastatin treatment. Some of the most frequently observed MMPs is MMP9, which plays a significant role in breast cancer tumor colonization, metastasis, and epithelial-to-mesenchymal transition [103]. Investigators studied the signatures of MMP9 in healthy and cancer breast tissue with various molecular subtypes [104] which showed a marked increase in the expression of MMP9 in cancer tissues relative to ordinary ones [105]. Additionally, it was discovered that MMP9 was differentially expressed within different molecular subtypes of breast cancer [104].

New findings suggest that two common features of tumors are alteration of the redox equilibrium and abolition of redox signals that are closely correlated with malignancy and drug resistance [106]. Therefore, it can be predicted that the upregulation of SOD, GSH, and CAT will contribute to a rise in H_2_O_2_ levels in the mitochondria, which is a major signaling molecule and a “reactive oxygen species” [107]. Several experiments have shown that mitochondrial H_2_O_2_ is a strong and efficient inducer of the apoptotic cycle [108]. Treatment with the ruthenium-fluvastatin complex substantially improved the production of SOD, CAT, and GSH in breast cancer, possibly through enabling ROS to induce apoptotic events.

Uncontrolled proliferation is a signature of tumors and can be analyzed using a variety of methods, including the counting of mitotic figures in stained tissue samples, the inclusion of labeled nucleotides in DNA, and the cytometric flow measurement of the percentage in the S stage of a cell cycle [109]. Dowsett determined that an immunohistochemical test of the Ki-67 antigen was one of the most important approaches for quantifying proliferation [110]. Ki-67 is predominant in all cancer cells, and its role as a proliferation predictor is of great importance. The proliferation biomarker Ki-67 has also been regarded as a diagnostic biomarker for breast cancer in many studies [111, 112]. Our analysis shows that the carcinogen control animals displayed a rise in the number of cells labeled with Ki-67 by decreasing AI, suggesting cell proliferation in the breast tissue. On the other hand, after treatment with the ruthenium-fluvastatin complex, a decrease in cells labeled with Ki-67 and consequent rise in AI were observed.

**Figure 15:**
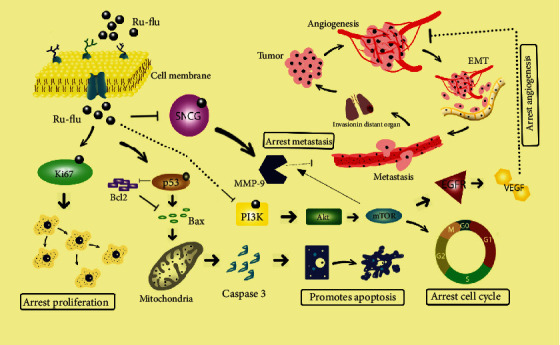
Probable molecular mechanistic pathway of the ruthenium-fluvastatin complex in the induction of apoptosis in breast cancer.

In conclusion, the ruthenium-fluvastatin complex is accountable for p53 interfering apoptosis in breast carcinoma, facilitated by the intrinsic apoptotic path provoked by Bcl2 and Bax and simultaneously regulating the PI3K/Akt/mTOR pathway in conjunction with MMP9-regulated invasive tumor pathways (Figure 15). Moreover, the complex reveals antiangiogenic functions by decreasing the EGFR and VEGF biomarkers as well. At the same time, the complex showed a high activity of its free radical scavenging potential in breast carcinoma cells caused by the release of reactive oxygen species extracted from mitochondrial through p53 regulation. The attenuation of Ki-67 coupled with stimulation of p53 further increases apoptosis attained by limiting cell proliferation. The observations offer ample proof that low doses of ruthenium-fluvastatin chemotherapy could interrupt, suspend, or delay breast carcinoma development by observing that the biomarkers correlated with the inhibition of apoptotic processes in breast carcinoma.

## 5. Conclusions

The current study concluded that the novel ruthenium-fluvastatin complex is capable of downregulating SNCG expression associated with breast carcinoma cell line regulation by inhibiting cell proliferation and inducing apoptosis by triggering the cascade of PI3K/Akt/mTOR/VEGF/MMP9. In conjunction, the complex was able to alter p53 expressions to interfere with apoptosis in breast carcinoma, induced by the intrinsic apoptotic pathway facilitated by Bcl2 and Bax and associated with MMP9-regulated tumor pathways at the same time regulating the PI3K/Akt/mTOR/VEGF pathway. Our evidence found that chemotherapy with ruthenium-fluvastatin can inhibit, reverse, or impede the advancement of breast carcinoma by altering intrinsic apoptosis as well as the antiangiogenic sequence, thus assuming the potential future role of a potential contender in cancer therapy.

## Figures and Tables

**Table 1:** Ligand with its binding energy values from docking studies.

Name	Electrostatic energy	Van der waals energy	Cdocker energy	Cdocker_interaction energy	Calculate binding energy	H-bond count	Interacting amino acids
Ruthenium-fluvastatin	-23.168	3.03	-234.9	45.31	-56.3119	2	Thr59,Asn64, Val66, Ser67, Glu68, Val71

**Table 2:** FTIR spectrum of fluvastatin and the ruthenium-fluvastatin complex (band position (cm^−1^)).

Compound	*ν* (O-H)	*ν* (C-N)	*ν* (C-F)	*ν* (C-O)	*ν* (C=C)	*ν* (M-O)
Fluvastatin	3376.46	1215.98	967.80	1105.21	1570.14	—
Complex	3412.27	1220.12	970.13	1098.12	1543.68	600.14

**Table 3:** Hematological finding in male Swiss albino mice treated with the ruthenium-fluvastatin complex for a 28-day repeated-dose oral subacute toxicity study.

Parameters (±SEM)	Control	Ru-flu complex (200 mg)	Ru-flu complex (100 mg)	Ru-flu complex (50 mg)	Ru-flu complex (25 mg)
Hemoglobin (%)	12.30 ± 0.015	13.44 ± 0.01	12.64 ± 0.01	12.25 ± 0.01	12.60 ± 0.02
Total RBC (×10^6^/*μ*)	4.27 ± 0.003	5.10 ± 0.001^#^	4.51 ± 0.001	4.33 ± 0.002	4.62 ± 0.001
Platelet count (×10^5^/*μ*)	2.85 ± 0.001	3.25 ± 0.03	2.55 ± 0.004	2.58 ± 0.001	2.78 ± 0.001
WBC (×10^3^/*μ*)	8.74 ± 0.02	13.21 ± 0.02^#^	11.73 ± 0.02	3.46 ± 0.01	6.4 ± 0.04
MCV (fL)	88.86 ± 0.01	91.34 ± 0.01	88.50 ± 0.03	89.85 ± 0.01	88.84 ± 0.01
MCH (pg)	28.44 ± 0.01	28.97 ± 0.09	28.14 ± 0.01	28.17 ± 0.03	28.13 ± 0.01
MCHC (%)	31.19 ± 0.02	31.75 ± 0.06	32.84 ± 0.01	32.26 ± 0.01	32.56 ± 0.02
Neutrophil (%)	28.59 ± 0.10	29.14 ± 0.02	25.24 ± 0.12	27.50 ± 0.15	27.18 ± 0.03
Eosinophil (%)	2.08 ± 0.03	6.08 ± 0.03	6.08 ± 0.03	5.20 ± 0.08	1.04 ± 0.01
Monocytes (%)	1.20 ± 0.08	2.10 ± 0.06	1.20 ± 0.08	1.20 ± 0.08	2.10 ± 0.06
Basophil (%)	0.0 ± 0.0	0.0 ± 0.0	0.0 ± 0.0	0.0 ± 0.0	0.0 ± 0.0

Standard error of mean = standard deviation (SD)/√total subject. Results are analyzed by the *t*-test and one-way ANOVA and confirmed by Dunnett's multiple comparison test. MCV: mean corpuscular volume; MCH: mean corpuscular hemoglobin; MCHC: mean corpuscular hemoglobin concentration; RBC: red blood cell; WBC: white blood cell. ^#^Significant difference at *p* < 0.05, when compared with the control group.

**Table 4:** Hematological finding in female Swiss albino mice treated with the ruthenium-fluvastatin complex for a 28-day repeated-dose oral subacute toxicity study.

Parameters (±SEM)	Control	Ru-flu complex (200 mg)	Ru-flu complex (100 mg)	Ru-flu complex (50 mg)	Ru-flu complex (25 mg)
Hemoglobin (%)	12.36 ± 0.024	13.36 ± 0.13	12.58 ± 0.02	12.14 ± 0.024	13.02 ± 0.17
Total RBC (×10^6^/*μ*)	4.38 ± 0.005	5.16 ± 0.24^#^	4.61 ± 0.004	4.42 ± 0.003	4.71 ± 0.003
Platelet count (×10^5^/*μ*)	2.85 ± 0.002	3.11 ± 0.005	2.53 ± 0.002	2.58 ± 0.002	2.78 ± 0.002
WBC (×10^3^/*μ*)	8.84 ± 0.03	13.36 ± 0.02^#^	11.88 ± 0.02	3.72 ± 0.03	5.14 ± 0.004
MCV (fL)	88.96 ± 0.02	91.34 ± 0.02	88.54 ± 0.05	89.06 ± 0.23	89.12 ± 0.23
MCH (pg)	28.16 ± 0.19	29.98 ± 0.03	28.04 ± 0.03	28.06 ± 0.02	27.64 ± 0.26
MCHC (%)	32.35 ± 0.044	32.79 ± 0.05	31.83 ± 0.05	31.41 ± 0.02	31.77 ± 0.03
Neutrophil (%)	28.72 ± 0.24	32.36 ± 0.05	24.92 ± 0.20	26.92 ± 0.20	24.12 ± 0.31
Eosinophil (%)	2.21 ± 0.09	2.27 ± 0.12	2.08 ± 0.04	3.08 ± 0.04	2.02 ± 0.02
Monocytes (%)	2.02 ± 0.02	2.32 ± 0.09	1.15 ± 0.04	1.25 ± 0.06	2.15 ± 0.01
Basophil (%)	0.0 ± 0.0	0.0 ± 0.0	0.0 ± 0.0	0.0 ± 0.0	0.0 ± 0.0

Standard error of mean = standard deviation (SD)/√total subject. Results are analyzed by the *t*-test and one-way ANOVA and confirmed by Dunnett's multiple comparison test. MCV: mean corpuscular volume; MCH: mean corpuscular hemoglobin; MCHC: mean corpuscular hemoglobin concentration; RBC: red blood cell; WBC: white blood cell. ^#^Significant difference at *p* < 0.05, when compared with the control group.

**Table 5:** Serum biochemistry findings in male Swiss albino mice treated with the ruthenium-fluvastatin complex for a 28-day repeated-dose oral subacute toxicity test.

Parameters (±SEM)	Control	Ru-flu complex (200 mg)	Ru-flu complex (100 mg)	Ru-flu complex (50 mg)	Ru-flu complex (25 mg)
AST	31.28 ± 0.12	50.01 ± 0.02^#^	40.05 ± 0.01	35.39 ± 0.07	32.15 ± 0.04
ALT	31.28 ± 0.08	50.06 ± 0.02^#^	37.54 ± 0.04	45.48 ± 0.07	34.08 ± 0.04
ALP	76.65 ± 15.25	103.7 ± 53.1^#^	78.8 ± 14.27	77.3 ± 11.80	76.5 ± 15.21
Total protein (g/dL)	6.22 ± 0.22	2.23 ± 0.26^#^	6.66 ± 0.15	6.14 ± 0.10	6.22 ± 0.14
Blood urea nitrogen (mg/dL)	18.10 ± 0.03	30.16 ± 0.02^#^	28.56 ± 0.02	28.44 ± 0.04	19.06 ± 0.02
Creatinine (mg/dL)	0.60 ± 0.002	0.60 ± 0.002	0.60 ± 0.002	0.55 ± 0.002	0.50 ± 0.004
Glucose (mg/dL)	115.2 ± 0.37	129.4 ± 0.40^#^	115.2 ± 0.37	115.7 ± 0.23	119.4 ± 0.40
Cholesterol (mg/dL)	47.10 ± 0.044	50.12 ± 0.05	47.08 ± 0.03	45.09 ± 0.04	43.06 ± 0.04

Standard error of mean = standard deviation (SD)/√total subject. Results are analyzed by the *t*-test and one-way ANOVA and confirmed by Dunnett's multiple comparison test. ^#^Significant difference at *p* < 0.05, when compared with the control.

**Table 6:** Serum biochemistry in female Swiss albino mice treated with the ruthenium-fluvastatin complex for a 28-day repeated-dose oral subacute toxicity study.

Parameters (±SEM)	Control	Ru-flu complex (200 mg)	Ru-flu complex (100 mg)	Ru-flu complex (50 mg)	Ru-flu complex (25 mg)
AST	31.18 ± 0.12	50.11 ± 0.02^#^	40.15 ± 0.01	35.49 ± 0.07	32.05 ± 0.04
ALT	31.38 ± 0.08	50.16 ± 0.02^#^	37.64 ± 0.04	45.38 ± 0.07	34.12 ± 0.04
ALP	76.42 ± 10.2	109.7 ± 12.36^#^	74.8 ± 13.27	73.3 ± 12.80	71.5 ± 15.21
Total protein (g/dL)	6.23 ± 0.12	2.31 ± 0.27	6.12 ± 0.15	6.24 ± 0.10	6.21 ± 0.15
Blood urea nitrogen (mg/dL)	18.25 ± 0.03	32.16 ± 0.02^#^	28.12 ± 0.02	28.75 ± 0.04	19.43 ± 0.02
Creatinine (mg/dL)	0.60 ± 0.002	0.61 ± 0.002	0.62 ± 0.002	0.55 ± 0.002	0.50 ± 0.004
Glucose (mg/dL)	112.2 ± 0.37	131.4 ± 0.40^#^	115.1 ± 0.37	117.7 ± 0.23	119.4 ± 0.40
Cholesterol (mg/dL)	47.20 ± 0.04	50.02 ± 0.05	47.18 ± 0.03	45.19 ± 0.04	43.16 ± 0.04

Standard error of mean = standard deviation (SD)/√total subject. Results are analyzed by the *t*-test and one-way ANOVA and confirmed by Dunnett's multiple comparison test. ^#^Significant difference at *p* < 0.05, when compared with the control group.

**Table 7:** Effect of ruthenium, fluvastatin, and the ruthenium-fluvastatin complex on the expression of Bax, Bcl2, p53, and MMP9 in breast tissues.

Groups	p53^§^	Bcl2^§^	Bax^§^	MMP9^§^
Control	8.5 ± 0.2	7.1 ± 0.7	7.9 ± 0.3	8.3 ± 0.1
DMBA	3.2 ± 0.8	15.2 ± 1.8	3.5 ± 0.7	19.5 ± 0.7
Ru-flu 25 mg/kg	6.7 ± 0.1	12.6 ± 0.3^∗∗^	4.1 ± 0.8^∗∗^	15.5 ± 0.2
Ru-flu 50 mg/kg	8.7 ± 0.2^∗∗^	9.6 ± 0.4^∗∗^	5.2 ± 0.7^∗^	12.7 ± 0.8^∗∗^
Ru-flu 75 mg/kg	9.3 ± 0.9^∗^	7.2 ± 0.9^∗^	8.3 ± 1.6^∗^	10.2 ± 0.1^∗^
Ru 50 mg/kg	6.4 ± 0.4	10.2 ± 0.5	4.5 ± 0.7	14.7 ± 0.2
Flu 50 mg/kg	5.5 ± 0.9	11.7 ± 1.2	4.9 ± 0.3	16.4 ± 1.6

^§^Each score represents the results of 6 slides per rat and 6 rats per group (mean ± SE, *n* = 6). Each field was selected randomly for evaluation of percentage of immune-positive cells. ^∗^Significant difference between the treated groups and carcinogen control (*p* < 0.01). ^∗∗^Significant difference between the treated groups and carcinogen control (*p* < 0.05).

**Table 8:** Cell proliferation and apoptosis in the breast.

Groups	Ki-67-LI^§^	AI (%)^§^	*R* = Ki‐67‐LI/AI
Normal control	21.08 ± 0.4	0.16 ± 0.02	131.75 ± 0.2
DMBA	38.9 ± 1.3	0.07 ± 0.02	555.714 ± 0.5
Ru-flu 25 mg/kg	26.3 ± 0.2	0.09 ± 0.05	292.222 ± 0.1
Ru-flu 50 mg/kg	22.8 ± 0.1^∗∗^	0.14 ± 0.02^##^	162.857 ± 0.2^$$^
Ru-flu 75 mg/kg	19.3 ± 0.6^∗^	0.16 ± 0.03^#^	120.625 ± 0.9^$^
Ru 50 mg/kg	24.8 ± 0.1	0.07 ± 0.02	354.245 ± 0.4
Flu 50 mg/kg	27.8 ± 0.6	0.11 ± 0.04	252.727 ± 0.2

LI = labeling index; Ki-67-LI = percentage of PCNA-labeled cells/total number of cells counted; AI = apoptotic index. *R* = PCNA‐LI/AI. AI was calculated as the percentage of TUNEL-positive cells/total number of cells counted. Values represent mean ± SE. ^§^A total number of six slides were evaluated per rat. Each field consisted of approximately 500 cells. ^∗^Significant difference between Ki-67-LI of Ru-flu 75 mg/kg and carcinogen control animals (*p* < 0.01). ^∗∗^Significant difference between Ki-67-LI of Ru 50 mg/kg, Ru-flu 50 mg/kg, and carcinogen control animals (*p* < 0.05). ^#^Significant difference between AI of Ru-flu 75 mg/kg and carcinogen control (*p* < 0.01). ^##^Significant difference between AI of Ru 50 mg/kg, Ru-flu 50 mg/kg, and carcinogen control animals (*p* < 0.05). ^$^Significant difference between *R* of Ru-flu 75 mg/kg and carcinogen control animals (*p* < 0.01). ^$$^Significant difference between R of Ru 50 mg/kg, Ru-flu 50 mg/kg, and carcinogen control animals (*p* < 0.05).

## Data Availability

The information used to validate the results of this study is included within the paper.
